# Cleavage of human tau at Asp421 inhibits hyperphosphorylated tau induced pathology in a *Drosophila* model

**DOI:** 10.1038/s41598-020-70423-1

**Published:** 2020-08-10

**Authors:** Hao Chi, Lee Sun, Ren-Huei Shiu, Rui Han, Chien-Ping Hsieh, Tzu-Min Wei, Chung-Chuan Lo, Hui-Yun Chang, Tzu-Kang Sang

**Affiliations:** 1 Institute of Biotechnology, National Tsing Hua University, Hsinchu, 30013 Taiwan; 2 Institute of Bioinformatics and Structural Biology, National Tsing Hua University, Hsinchu, 30013 Taiwan; 3 Institute of Systems Neuroscience, National Tsing Hua University, Hsinchu, 30013 Taiwan; 4 Brain Research Center, National Tsing Hua University, Hsinchu, 30013 Taiwan; 5 Department of Medical Science, National Tsing Hua University, Hsinchu, 30013 Taiwan; 6 Department of Life Science, National Tsing Hua University, Hsinchu, 30013 Taiwan

**Keywords:** Genetics, Neuroscience, Diseases, Pathogenesis

## Abstract

Hyperphosphorylated and truncated tau variants are enriched in neuropathological aggregates in diseases known as tauopathies. However, whether the interaction of these posttranslational modifications affects tau toxicity as a whole remains unresolved. By expressing human tau with disease-related Ser/Thr residues to simulate hyperphosphorylation, we show that despite severe neurodegeneration in full-length tau, with the truncation at Asp421, the toxicity is ameliorated. Cytological and biochemical analyses reveal that hyperphosphorylated full-length tau distributes in the soma, the axon, and the axonal terminal without evident distinction, whereas the Asp421-truncated version is mostly restricted from the axonal terminal. This discrepancy is correlated with the fact that fly expressing hyperphosphorylated full-length tau, but not Asp421-cleaved one, develops axonopathy lesions, including axonal spheroids and aberrant actin accumulations. The reduced presence of hyperphosphorylated tau in the axonal terminal is corroborated with the observation that flies expressing Asp421-truncated variants showed less motor deficit, suggesting synaptic function is preserved. The Asp421 cleavage of tau is a proteolytic product commonly found in the neurofibrillary tangles. Our finding suggests the coordination of different posttranslational modifications on tau may have an unexpected impact on the protein subcellular localization and cytotoxicity, which may be valuable when considering tau for therapeutic purposes.

## Introduction

Tauopathies are a group of neuropathological conditions commonly found in patients with Alzheimer’s disease (AD) and various forms of parkinsonism. Human tau protein is encoded by the *MAPT* (microtubule-associated protein tau) gene. Through alternative splicing of exons 2, 3, and 10, this gene encodes six different isoforms, with either three or four microtubule-binding domains. Besides an apparent role in microtubule binding and stabilization, tau can interact with actin filament and cell membrane, and may mediate signal regulation^1–3^. The longest isoform, 2N4R tau (composed of 441 amino acids, also known as 2N4R), is widely used in the study of tau-induced pathogenic mechanisms^4^.

Studies from patients and animal models have demonstrated that aberrant posttranslational modifications (PTMs) of tau, especially hyperphosphorylation, prevents the protein from binding and stabilizing microtubules^5^. Instead, the modified proteins form aggregates, which impair a range of neuronal functions, including neurotransmission and actin organization^6,7^, which eventually lead to neurodegeneration^4^. It has been noted that cleaved tau variants are present in the aggregates and are associated with diseases^8^. Therefore, different tau PTMs, including phosphorylated modifications and proteolytic truncations, may play a critical role in the pathogenesis of tauopathies.

Wild-type tau protein is unstructured, and PTMs can affect its folding, protein interaction, and subcellular localization. Such modifications are dynamic and vary with different physiological and pathological conditions^9^. Indeed, 2N4R tau has 97 Ser, Thr, Tyr, and His residues that can potentially be phospho-modified by a panel of kinases. Hyperphosphorylated tau tends to form paired helical filaments (PHFs), the main constituent of neurofibrillary tangles^10,11^, which, together with amyloid-β, serve as the pathological hallmarks of AD^12^.

Although it is widely accepted that hyperphosphorylated tau is prone to aggregate formation and is pathogenic, a recent trial of tideglusib, a compound that targets the major tau kinase GSK-3β, failed to show clinical benefits in patients with AD^13^, raising the question of whether hyperphosphorylated tau is the sole cytotoxic source in AD. Furthermore, tau phosphorylation at specific residues can ameliorate rather than aggravate toxicity^14^, which is consistent with the fact that phosphorylated tau residues are widespread under normal physiological conditions^9^. Current knowledge of phosphorylated tau dynamics and the resulting functional effects is incomplete. The modulation of cdk5/p35 kinase did not impact human tau toxicity in a *Drosophila* model^15^, and a confounding study showed that the expression of mitogen-activated protein kinase p38γ could ameliorate tau toxicity through the phosphorylation of T205, a site that is also targeted by GSK-3β^14^. Therefore, whether tau hyperphosphorylation exerts cytotoxic effects remains an open question^14–18^.

Analyses of tau protein from AD brains revealed several truncated forms with cleavage sites at D13, D25, N368, E391, and D421 of 2N4R tau. Among these, tau isoforms C-terminally truncated at either E391 or D421 are enriched in neurofibrillary tangles and correlated with AD progression^8,19,20^. Tau truncation at D421, mediated by caspase-3/6, may promote self-aggregation, tangle formation, tau secretion, and neurotoxicity, highlighting the pathological significance of this truncated form^21–23^. Using human 0N4R tau, a *Drosophila* tauopathy model showed that the expression of D421-truncated isoform is more toxic than wild-type^24^. However, a study of tau transgenic mice showed that while caspase activation generates tau-D421-cleaved variant and tangle formation, those neurons remain alive^25^. Importantly, neurons exhibited truncated tau also showed increased phospho-epitope labeling^25^, suggesting the interaction of these two modes of PTM impact tau toxicity^18,26^.

Axonal spheroid is a prominent pathology of axonopathy that has been frequently observed in AD brains and mouse models overexpressing APP^27–29^. This aberrant structure precedes axonal disintegration and impairs cargo transport mediated by kinesin and dynein^30^. Axonal spheroid may associate with axonal actin aggregation as an actin stabilization agent can suppress the spheroid formation^31^. Hirano body is another common pathology found in different types of neurodegenerative conditions. This actin-based structure may contain tau, amyloid precursor protein, and other stress proteins^32–35^. A study showed that expressing hyperphosphorylated tau could induce actin rods similar to Hirano bodies in the *Drosophila* brain^7^. However, despite a close tie to the changes of the cytoskeleton, little is known about the underlying cause that regulates these two pathological features in the degenerating neurons.

Modeling human tau-induced cytotoxicity in *Drosophila* has yielded several landmark findings^7,36–39^. Here, we use this animal model to study the pathogenic impact of disease-associated tau hyperphosphorylation and cleavage-resulted tau D421-truncation^38,39^. To compare the cytotoxic effect of hyperphosphorylation and truncation, we generated a panel of transgenic flies bearing phospho-modified 2N4R tau and tau-D421 isoforms to directly compare the combinational effects of these two modes of modifications commonly observed in tauopathies. Our results support that cleavage of full-length tau at D421 ameliorates the cytotoxicity induced by hyperphosphorylated tau, suggesting that the interplay between different PTMs in tau protein may strongly impact its toxicity.

## Methods

### DNA constructs

To construct the cDNAs of normal human 2N4R tau, or with the modifications of Thr111, Thr153, Ser175, Thr181, Ser199, Ser202, Thr205, Thr212, Thr217, Thr231, Ser235, Ser396, Ser404, and Ser422 mutated to Ala (AP) or Glu (E14), we performed PCR using Phusion High-Fidelity DNA Polymerase (New England Biolabs) to amply DNA fragments from the genome of UAS transgenic flies bearing human 0N4R tau of AP and E14^7,38,39^, and from the plasmid with 2N4R tau cDNA. Primers 5′-ATGGCTGAGCCCCGCCAG-3′ and 5′-TGTGGTTCCTTCTGGGATCTCCG-3′ were used to amplify 1–306 of 2N4R tau cDNA. Primers 5′-GCTGAAGAAGCAGGCATTGGAGAC-3′ and 5′-TCACAAACCCTGCTTGGCCAGGG-3′ were used to amplify 133–1,152 of 0N4R tau cDNA with either E14 or AP mutations. The two fragments were ligated by T4 ligase (New England Biolabs) and subsequently purified. The purified product was subject to the second round of PCR using Taq DNA polymerase (New England Biolabs) with primers 5′-ATGGCTGAGCCCCGCCAG-3′ and 5′-TCACAAACCCTGCTTGGCCAGGG-3′. The constructs were then cloned into pCR2.1-TOPO vector using TOPO TA cloning kit to yield E14FL/APFL constructs before subcloned into pUAST attB vector for site-specific (attP-2) integration into the fly genome. The same approach was used to generate E14421/AP421 constructs, except that primers 5′-GCTGAAGAAGCAGGCATTGGAGAC-3′ and 5′-TCAGTCTACCATGTCGATGCTGCCG-3′ were used to amplify 133–1,089 of 0N4R tau cDNA with either E14 or AP mutations. Primers 5′-ATGGCTGAGCCCCGCCAG-3′ and 5′-TCAGTCTACCATGTCGATGCTGCCG-3′ were used to amplify 1–1,263 of 2N4R tau cDNA to generate WT421 constructs. For D421A constructs, site-direct mutagenesis primers 5′-AGCATCGACATGGTAGCCTCGCCCCAGCTCGCC-3′ and 5′-GGCGAGCTGGGGCGAGGCTACCATGTCGATGCT-3′ were used to introduce D421A mutation on the cDNA of E14FL and APFL. All modified human 2N4R tau cDNAs were sequencing confirmed.

### Fly genetics

Flies were maintained at 25 °C and raised on the standard cornmeal food under a 12:12-h light/dark cycle. The driver lines, *GMR-Gal4* and *GAD-Gal4*, have been described previously^40,41^. The *202508-Gal4* line was obtained from the Vienna Drosophila Resource Center. *UAS-mCD8-GFP*, *UAS-lacZ*, *UAS-Denmark*, *UAS-syt-GFP*, *tubP-Gal80*^*ts*^ and *UAS-shibire*^*ts*^ were obtained from the Bloomington Drosophila Stock Center.

### Subcellular fractions

The collection of protein extracts from subcellular fractions was based on previously published methods with modifications^36,42,43^. Fly heads were collected and homogenized in sucrose buffer (320 mM sucrose, 4 mM HEPES, pH 7.4, protease inhibitor cocktail, and PhosStop phosphatase inhibitor cocktail (Roche). The homogenate was centrifuged at 1,000 g for 10 min. The pellet, mainly containing the nuclei and high-density cellular components, was resuspended in RIPA buffer (150 mM NaCl, 1.0% IGEPAL CA-630/NP-40, 0.5% sodium deoxycholate, 0.1% SDS, 50 mM Tris, pH 8.0) and collected, and the supernatant was centrifuged at 15,000 g for 15 min. The second pellet, mainly containing low-density cellular components, was resuspended in a sucrose gradient solution consisting of 0.85, 1, and 1.2 M sucrose diluted in 5 mM Tris–HCl and centrifugated at 85,000 g for 2 h^43^. The 1 and 1.2 M sucrose interface contains synaptosomes were resuspended in 1 mM Tris–HCl and collected.

### Sarkosyl extraction

The sarkosyl extraction method is based on the previous publication^44^. The fly heads were collected and homogenized in the extraction buffer (25 mM Tris–HCl, pH 7.4, 150 mM NaCl, 1 mM EDTA, 1 mM EGTA, 5 mM Na_4_P_2_O_7_, 10 mM glycerophosphate, 30 mM NaF, 2 mM Na_3_VO_4_, 1 mM PMSF, and 10 μg/ml leupeptin, aprotinin, and pepstatin). The homogenate was centrifuged at 80,000 g at 4 °C for 15 min. The supernatant was discarded and the pellet was resuspended in A68 extraction buffer (10 mM Tris–HCl, pH 7.4, 0.8 M NaCl, 10% sucrose, 1 mM EGTA, 1 mM PMSF, and 10 μg/ml leupeptin, aprotinin, and pepstatin). After adding 1% sarkosyl and incubating at room temperature for 1.5 h, samples were centrifugated at 80,000 g for 30 min at 4 °C. The supernatant was collected as sarkosyl-soluble fraction, and the pellets were resuspended in 50 mM Tris–HCl and collected as the sarkosyl-insoluble fraction.

### Western blotting

Heads from adult flies were collected and homogenized in lysis buffer (10 mM Tris–HCl, pH 7.4; 150 mM NaCl; 5 mM EDTA; 5 mM EGTA; 10% glycerol; 50 mM NaF; 1 mM Na_3_VO_4,_; 5 mM NaPPi; 5 mM DTT; 4 M urea with protease inhibitor cocktail) at 4 °C. Protein extracts were centrifuged at 3,000 g for 3 min at 4 °C, and the supernatants were stored at -20 °C. Lambda phosphatase (New England Biolabs) was added to the thawed protein extracts and incubated at 30 °C for 30 min. Western blotting was performed following the standard procedure^45^. The primary antibodies were used with the following dilutions: rabbit anti-human pan tau (Dako, 1:20,000), mouse anti-tau-C3 (Invitrogen, 1:10,000), mouse anti-α-tubulin (GeneTex, 1:5,000), mouse anti-β-tubulin (Developmental Studies Hybridoma Bank, 1:5,000), mouse anti-AT8 (Thermo, 1:500), mouse anti-AT100 (Thermo, 1:500), mouse anti-ATP5a (Abcam, 1:100,000), rabbit-histone H3 (Abcam, 1:5,000), and mouse anti-syntaxin (Developmental Studies Hybridoma Bank, 1:5,000). Secondary antibodies conjugated with HRP (Jackson ImmunoResearch Laboratories) were used in 1:10,000 dilutions. All loading controls were prepared by stripping off the reagents from the original membrane and then re-immunoblotting following the standard procedures. Semiquantitative analysis of band density was performed in ImageJ.

### Immunocytochemistry

All flies were age-matched for experiments. The whole-mount preparation of fly eyes and brains was performed as previously described^46^.The following primary antibodies were used with the indicated dilutions: anti-lamin B1 (Sigma, 1:20), anti-GABA (GeneTex, 1:200), anti-human pan-tau (Dako, 1:200), anti-tau-C3 (Invitrogen, 1:200), anti-GFP (Abcam, 1:100), anti-AT8 (Thermo, 1:200), anti-AT100 (Thermo, 1:100), and anti-PHF1 (Abcam, 1:100). Alexa Fluor 488, Cy3, and Cy5 conjugated secondary antibodies (Jackson ImmunoResearch Laboratories) were used at 1:100 dilutions. F-Actin enriched rhabdomere and spots of aberrant actin accumulations were labeled by rhodamine-conjugated phalloidin (Sigma, 1:20) and Alexa Fluor 633-conjugated phalloidin (Thermo, 1:50), respectively. Samples were analyzed on Zeiss LSM510 or LSM800 confocal microscopes.

### Fly behavior

Flies were rendered unconscious before testing by cooling them on ice for 30 min, and then half of each wing was cut off to prevent flight. For *GAD* > *shi*^*ts*^ and corresponded controls, flies were heat-shocked at 30 °C for 2 h before anesthesia. During testing, each fly was placed individually for 1 min in a lighted circular arena (height:125 mm, diameter: 200 mm) surrounded by water, similar to that used in a previous study^47^. The arena was composed of 20 panels, with each panel containing four 8 × 8 matrices of light-emitting diodes (LEDs). The wavelength of the LED light was 572 nm to reduce the possibility of the different light spectrum may interfere locomotion. The LED lights were controlled by an Arduino MEGA 2560 board connected to a computer. A charge-coupled device (CCD) camera was connected to a computer to track the fly’s path. The tracking frame rate was approximately 12–15 frames per second; the location of the fly was encoded as X and Y values, and the motor function of the fly was analyzed with a Python script. The following indexes were used in behavior analyses. Walking distance: the total distance the fly moved in the arena during the recording. Speed: the mean speed of the fly when walking, but not when standing or wobbling. Wobbling: the percentage of time the fly spent moving only distances of 1 mm or less between each tracking frame. Each experiment tested at least 20 flies for each genotype and repeated three times.

### Image analysis

To quantify the eye phenotype, the orientation of phalloidin-stained photoreceptors from each unit eye, or ommatidia, were marked by drawing the trapezoid vectors^15^. The vector of each ommatidium in wild-type points in the same direction. The percentage of mis-orientation ommatidium was calculated by dividing the total number of ommatidia from each confocal image.

Brain images were analyzed in ImageJ (https://rsb.info.nih.gov/ij). To quantify the axonal spheroid number, the regional average of CD8-GFP intensity from the antennal lobe was measured. A threshold was set at two-folds of the average intensity to extract signals of potential axonal spheroids. We used the edge-finding, followed by the particle-counting functions to count the number of axonal spheroids. The criterion of an axonal spheroid was set to include signals only covering an area larger than 1.5 μm^2^, and granularity was set at 0.89–1.0 to ensure that the extracted areas were spheroid^27^. The spots of aberrant actin accumulations were counted similarly as the axonal spheroids, except that the threshold was set at 1.5-folds of the average regional signal intensity. The criterion was set to include only signals cover an area larger than 1 μm^2^, and granularity between 0.89–1.0.

For the colocalization ratios of tau protein with synaptotagmin-GFP, partial projection views of antennal lobes correspond to 5 µm thickness were used. Quantification was conducted using the colocalization finder plugin of ImageJ.

## Results

### Both hyper- and hypophosphorylated tau disrupted retinal structure of which phenotype could be rescued by D421 truncation

**Figure 1:**
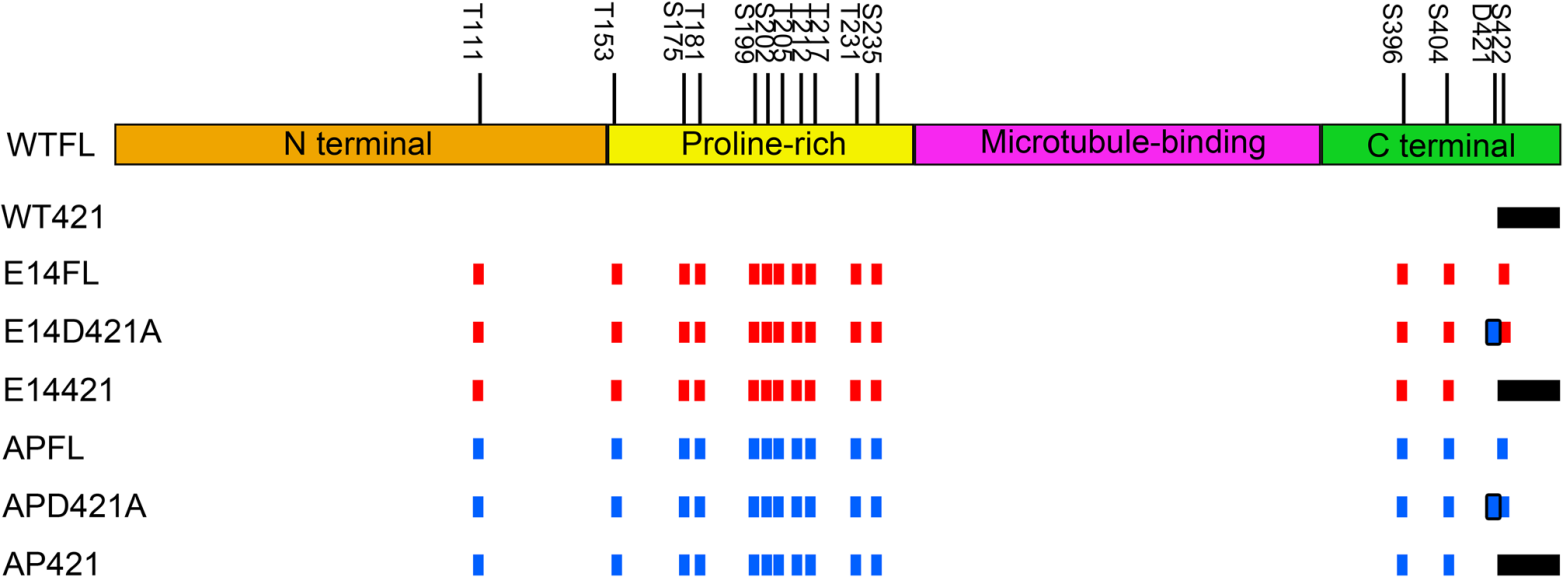
Schematic diagram of human tau isoforms used in the current study. Constructs of transgenic flies include WTFL, WT421, E14FL, E14D421A, E14421, APFL, APD421A, and AP421. WT: wild type; E14: 14 Ser/Thr sites as labeled, change to glutamic acids mimicking pseudo-phosphorylation as indicated by red bars. AP: the identical Ser/Thr residues as in E14 change to alanines mimicking non-phosphorylation as indicated by blue bars. FL: 1–441 residues; 421: 1–421 residues mimicking FL truncated at Asp421 site as indicated by the black column; D421A: Asp421 changes to alanine mimicking non-cleavage at Asp421 as indicated by the blue bars with black outlines; The different domains of tau are color-coded (orange: N terminal domain; yellow: the proline-rich domain; magenta: the microtubule-binding domain; green: C terminal domain).

**Figure 2:**
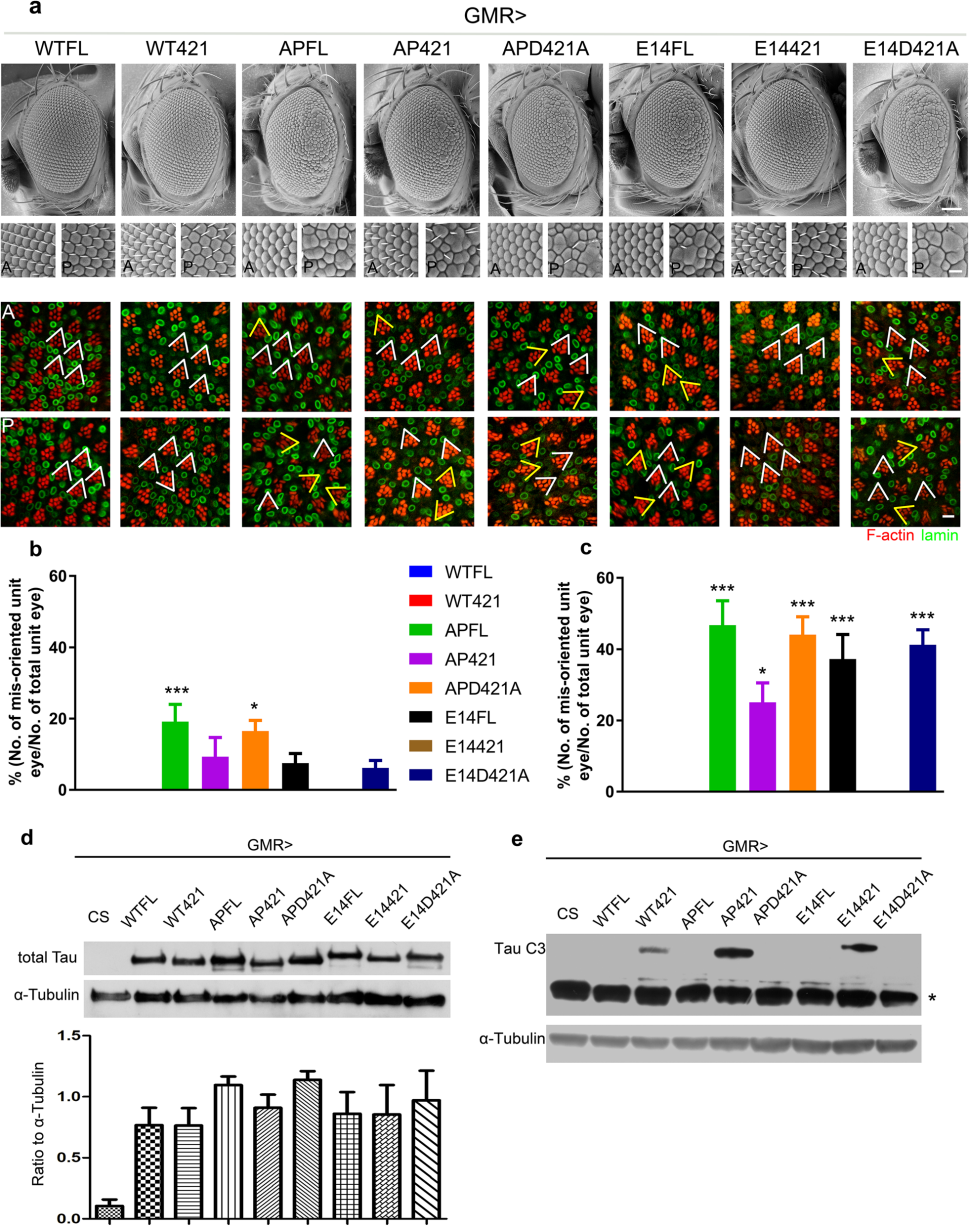
Cleavage of tau at D421 suppresses both tau hyperphosphorylation and hypophosphorylation induced toxicity in fly eye models. (**a**) Scanning electron microscopy (SEM) images of the compound eyes expressing the indicated tau isoforms under the control of *GMR-Gal4*; the lower SEM panels below each image are magnified views of the anterior (A) and posterior (P) areas of the corresponded eye. Confocal images of retinas from the corresponded genotypes stain with phalloidin (red) and anti-lamin (green) antibody to visualize the rhabdomeres and the nuclear envelope of the anterior (A) and posterior (P) part of the eye, respectively. The V-shaped white lines mark the orientation of the trapezoid organization of the photoreceptors in a unit eye. All images are taken from 1-day-old adult flies. Scale bars: 100 μm (whole eye SEM), 10 μm (magnified SEM panels), 5 μm (confocal images). (**b-c**) Quantification of mis-orientated unit eyes in the anterior (**b**) and the posterior (**c**) areas of the indicated tau isoforms. V-shaped yellow lines mark the mis-orientated unit eyes. Six eyes from 1-day-old flies of each isoform are examined. Values shown represent mean ± SE. *p < 0.05, ***p < 0.001 (one-way ANOVA with Bonferroni multiple comparison test). (**d, e**) Western analysis of tau protein (**d**), and D421 truncated tau protein (**e**) levels in tissues expressing the indicated tau isoforms under the control of *GMR-Gal4*. CS (Canton-S) is the wild type fly with no human tau expression. The asterisk in (**e**) indicates a non-specific band. The blots are stripped and re-probed with α-Tubulin to serve as a loading control. The ratio values are calculated as “total Tau/α-Tubulin” (**d**, n = 3, one-way ANOVA with Bonferroni multiple comparison tests shows no significant difference among tau isoforms), and “Tau C3/α-Tubulin” (**e**) respectively. The images of (**d**) are cropped from the same blot, and the full-length blot refers to Figure S5. The images of (**e**) are cropped from the same blot, and the full-length blot refers to Figure S6. Protein lysates are extracted from 1-day-old flies.

To resolve the possible interplay between phospho-modifications and the truncation of tau in vivo, we used human 2N4R tau (hereafter referred to as tau) with 14 clinically relevant mutations of Ser/Thr residues to mimic tau hyper- or hypophosphorylation, and a caspase cleavage site at D421 to imitate a commonly reported truncation (Fig. 1). The transgenic constructs were cloned into the pUAS-attB vector to ensure site-specific insertion into the attB-site on chromosome 3, causing the transgenes to be expressed at comparable levels (Fig. 2d). We first evaluated the cytotoxicity of different tau constructs using the *GMR-Gal4* driver to express the transgenes in the eye, which has a highly organized structure that enables easy comparison. We observed that neither wild-type full-length nor wild-type truncated tau caused detectable eye phenotype. Conversely, both hyperphosphorylated full-length tau (E14FL) and hypophosphorylated full-length tau (APFL) resulted in a rough eye phenotype on the posterior part of the eyes (Fig. 2a).

Expression of wild-type full-length tau (WTFL) by *GMR-Gal4* driver failed to induce rough eye phenotype, unlike previous reports using the same system to express wild-type 0N4R tau (WT0N4R)^38,39^. On the contrary, APFL in our model showed the rough eye phenotype, but the 0N4R counterpart did not unless with a higher expression level^38^. To solve such disparity, we used the same *GMR-Gal4* to express wild-type, E14, and AP, from the corresponded 0N4R and 2N4R transgenic lines^38,39^. For wild-type, the result of quantitative Western blotting showed a ~ threefold increase of protein levels in 0N4R as compared to 2N4R, which may explain the rough eye phenotype observed in the previously published 0N4R, but not in 2N4R generated here (see Supplementary Fig. S1a and S1b online). Moreover, the Western blotting result showed a large portion of the wild-type 0N4R tau proteins appeared to be phosphorylated as the phosphatase-treatment could lower the shifted proteins to the expected size, consistent with the original report^38^ (see Supplementary Fig. S1c online). For E14 and AP, with the comparable expression levels between each of their own 0N4R and 2N4R tau versions, the mild eye roughness could be found from 0N4R/2N4R E14FL, as well as from 0N4R/2N4R APFL (see Supplementary Fig. S1a and S1b online). Together, the discrepancy of eye phenotype between 0N4R and 2N4R wild-type tau variants is likely due to the level of protein expression and the state of phosphorylation, although we could not exclude that the N-terminus of tau might have a role in modulating cytotoxicity.

The eye roughness of 2N4R tau variants corresponded to disruption of the photoreceptor organization inside the eye, suggesting that the collective phosphorylation or dephosphorylation of those 14 Ser/Thr residues could render wild-type tau toxic (Fig. 2a–c). Remarkably, in comparison to E14FL and APFL, flies expressing the truncated form of E14421 showed essentially normal eye morphology, and that AP421 was also less toxic compared to APFL (Fig. 2a). Moreover, the disrupted internal cellular organization found in the corresponding full-length constructs was largely reversed, with E14421 reminiscent of the wild-type (Fig. 2b, c). These data suggest that truncation at D421 can suppress tau-induced toxicity.

Because E14FL and APFL may undergo cleavage at D421 by endogenous caspases, in which case would undermine the argument that truncation rescues the phenotype. However, such endogenous truncation, as assayed by the tau C3 antibody specific to D421-cleaved tau, was not detected (Fig. 2e). We tested this possibility further by expressing full-length phosphorylated/dephosphorylated constructs in which the D421 residue was replaced by Ala (E14D421A and APD421A), rendering these constructs insensitive to endogenous caspase cleavage. These caspase cleavage-insensitive constructs displayed a disrupted retinal phenotype similar to that observed with the original E14FL and APFL constructs (Fig. 2a–c), further proving that E14FL and APFL do not undergo endogenous truncation. Together, these results suggest that phosphorylation or dephosphorylation of tau at the 14 targeted residues converts tau into a toxic species, but that truncation at the D421 residue protects against toxicity.

### D421 cleavage suppresses tau-induced neurotoxicity and motor deficits

Because tauopathies mainly affect the central nervous system (CNS), we decided to test whether the observed protective effect of D421 truncation in the fly retina could be recapitulated in the brain. GABAergic neurons have been implicated in tauopathies, so we tested the expression of different tau constructs under the control of pan-GABAergic driver *GAD-Gal4*^41,48–51^. To test for a basic functional link between GABA activity and behavior, we analyzed fly motion behavior in a custom-made arena equipped with digital recording and automated movement analysis. When we expressed a temperature-sensitive shibire transgene (shi^ts^) to block neurotransmission of GAD neurons (*GAD* > *shi*^*ts*^), we observed movement defects compared with *GAD* > *lacZ* controls and the *GAD-Gal4* driver alone: the *GAD* > *shi*^*ts*^ flies were less active, with a reduced moving speed, and spent more time wobbling [see Supplementary Fig. S2 online]. Inhibition of GABA production by silencing GAD1 gene could cause locomotion defects in *Drosophila*^52^, which is consistent with our finding that a functional deficit in GAD neurons affects motor coordination.

**Figure 3:**
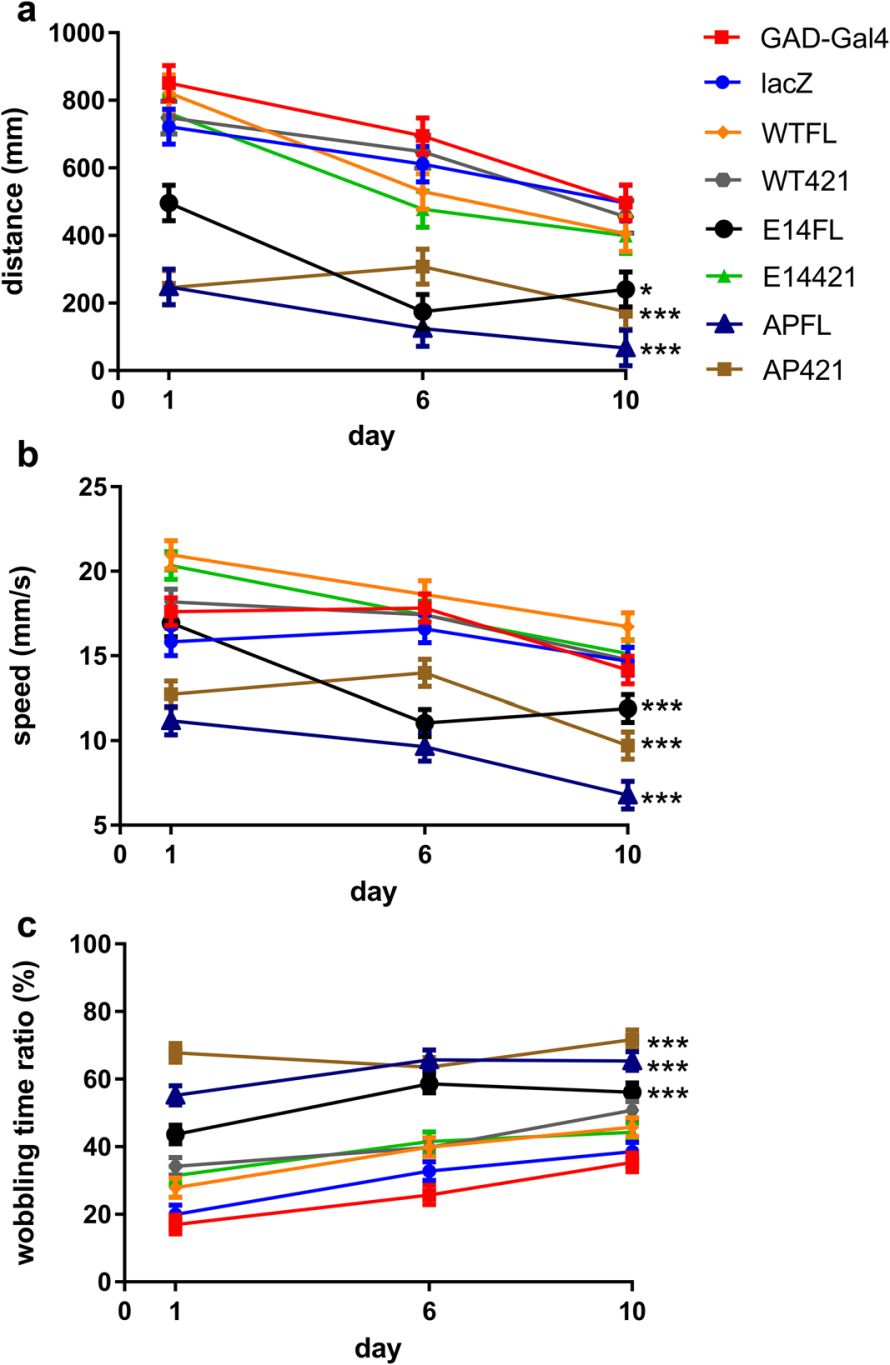
D421 truncation alleviates the motor impairments induced by phospho-modified tau. Analyses of motor behaviors from flies expressing the indicated tau isoforms and lacZ control driven by *GAD-Gal4*. The index of each analysis includes distance (**a**), speed (**b**), and wobbling time ratio (**c**), should refer to Methods for detail. At least 20 flies from three different ages (1-day, 6-day, 10-day) of each genotype were tested. Values shown represent mean ± SE. *p < 0.05, ***p < 0.001 (two-way ANOVA with LSD post hoc tests).

When testing flies expressing different tau constructs, we also observed profound behavioral deficits in *GAD* > *APFL, GAD* > *AP421* and *GAD* > *E14FL* flies. The motor activity of these flies was markedly reduced at the youngest age tested (1-day-old), and continued to decline as the flies aged. In contrast, *GAD* > *WTFL, GAD* > *WT421* and *GAD* > *E14421* flies showed similar motor activity to the lacZ control and GAD driver alone (Fig. 3a–c). These data strengthened the association between cytologic and behavioral phenotypes. Because the hyperphosphorylation of tau links to tauopathy and is clinically relevant, we focused on the comparison between E14FL and E14421 in the following assays.

### Tau truncation at D421 does not affect the protein stability

A previous study showed that the truncation of full-length tau at D421 could change its degradation pathway in vitro^53^. To determine whether E14421 is less stable than E14FL, and thus less cytotoxic, first we compared the protein levels of E14421 and E14FL at different stages using Western blots. Flies expressing WTFL, E14FL, and E14421 all showed higher levels of tau protein in 10-day-old brain lysates than in 1-day-old brain lysates (see Supplementary Fig. S3a and S3b online), but the protein levels among different constructs did not show significant differences at the ages tested.

Next, to directly address if there is any difference in degradation speed between E14FL and E14421 or WTFL and WT421, we adopted the inducible expression system using temperature-sensitive *Gal80*^*ts*^ to control Gad-Gal4-driven expression of tau protein and examined the protein levels by Western blotting. The flies were incubated at 18 °C throughout the development, and the adult flies were transferred to a 30 °C water bath for two days to induce tau expression. One set of flies was subject to protein extraction after the heat shock immediately, and the other set was placed back to 18 °C incubator for ten days before sacrifice. We found even after returning to the permissive temperature of Gal80 for ten days, tau protein levels remained high among tested variants as compared to those right after induction, except for WTFL, which instead had a 25–30% increase after the recovery (see Supplementary Fig. S3c and S3d online). We suspect this occurrence may due to the leaky expression of the basal heat shock promoter on the UAS constructs after shifting from 30 °C back to 18 °C^54,55^, and likely the slow degradation of human tau in *Drosophila*. Therefore, the previously reported effects of D421 truncation on protein degradation in vitro do not appear to be a major factor underlying the severity of the cytotoxic effect. Together, our data from *Drosophila* suggests that cleavage of tau at D421, a variant commonly found in lysates of tauopathy brains, can reduce tau-induced cytotoxicity in vivo.

### Tau truncation at D421 alters the distribution of tau protein in neurons

**Figure 4:**
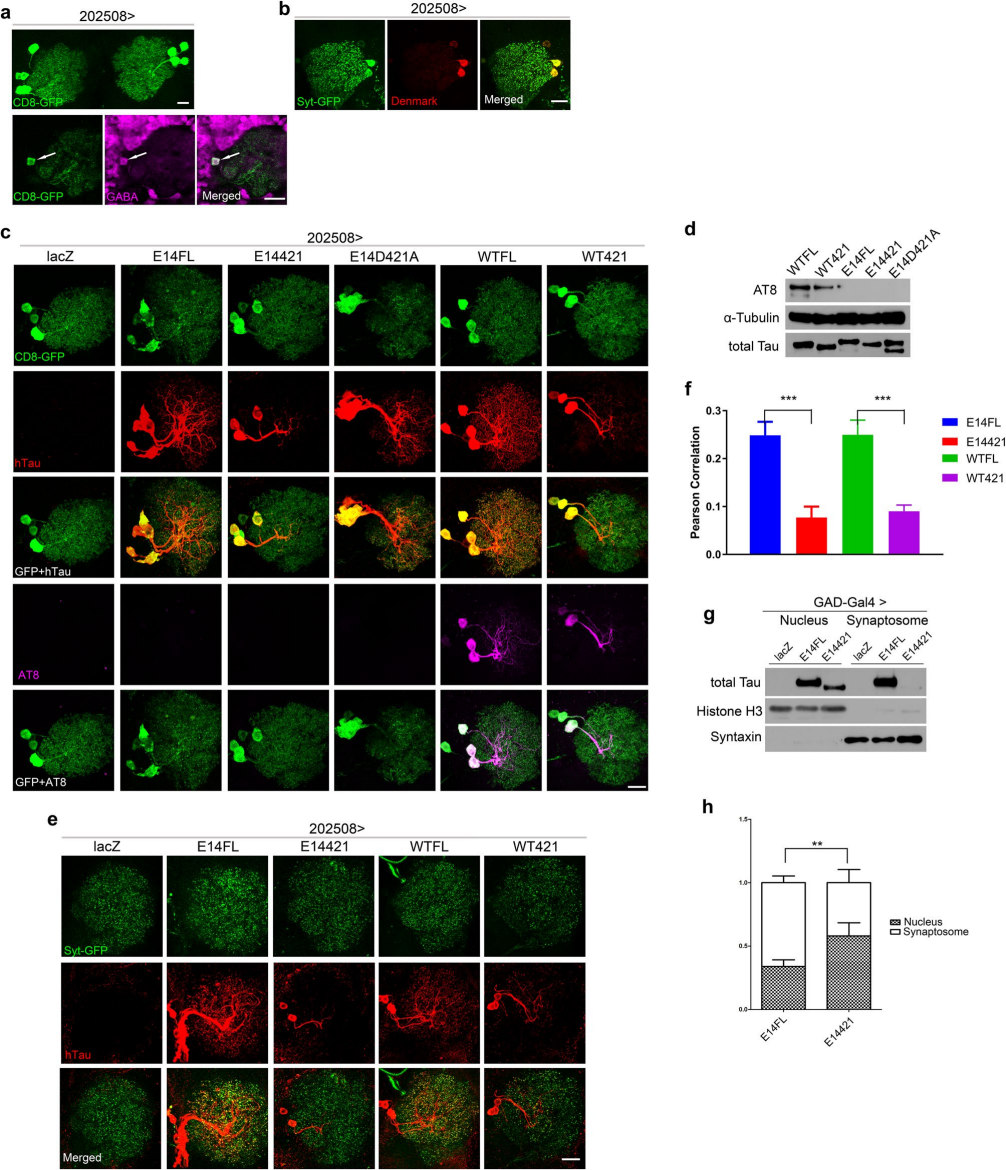
D421 cleavage affects the cellular distribution of hyperphosphorylated tau. **(a–c)** Confocal images of (**a**) neurons mark by membrane-tethered GFP driven by *202508-Gal4* (*202508* > *CD8-GFP*, upper panel), and with anti-GABA staining (magenta, lower panels). Arrows indicate the cell body of a 202508-expressing neuron. (**b**) *202508-Gal4* drives the expression of synaptic marker Syt-GFP and dendritic marker Denmark to label axons (green) and dendrites (red), respectively. (**c**) Immunolabeling of anti-hTau (red) and anti-AT8 (purple) from the brains of *202508-Gal4* driving lacZ and the indicated tau isoforms with the co-expression of CD8-GFP. Images are taken from 1-day-old adult flies. (**d**) Western analysis of phospho-tau protein (AT8) levels from 1-day-old fly heads of the indicated tau isoforms under the control of *GAD-Gal4*. The blots are stripped and re-probed with α-Tubulin, then stripped and re-probed with total tau to serve as loading controls. The images are cropped from the same blot, and the full-length blot refers to figure S11. (**e**) Confocal images of immunolabeling of anti-hTau (red) from the brains of *202508-Gal4* driving lacZ and the indicated tau isoforms with the co-expression of syt-GFP. Images are taken from 10-day-old adult flies. Scale bars: 10 μm (**a**, upper panel), 20 μm (**a**, lower panels), 20 μm **(b)**, 10 μm **(c, e)**. (**f**) Quantification of Syt-GFP and tau colocalization, as shown in (**d**). Six brains are measured for each genotype. See “Methods” for the detail of Pearson correlation analysis. All the examined flies were 10-day-old (n = 6, ***p < 0.001, one-way ANOVA with Bonferroni multiple comparison tests). **(g)** Western blot analysis of GABA neurons expressing lacZ (control) and the indicated tau isoforms with a *GAD-Gal4* driver. Different fractions of neuronal extracts, including nucleus/high-density cellular components and synaptosomes, are separated for analysis. Syntaxin is a marker of synaptosome, and histone H3 is a marker of nucleus. The blots are stripped and re-probed with syntaxin and histone H3 sequentially to serve as loading controls. The images are cropped from the same blot, and the full-length blot refers to Figure S12. **(h)** Quantification of replicated results shown in (**f)** the relative ratio of tau in each fraction is calculated as following: R1 = nucleus tau/histone H3, R2 = synaptosome tau/syntaxin; tau% in the nucleus = R1/(R1 + R2), tau% in the synaptosome = R2/(R1 + R2), **p < 0.01 (n = 5, two-way ANOVA with Bonferroni multiple comparison tests).

The broad-scale assays in the visual and the pan-GABA neurons support that D421 cleavage can reduce cytotoxicity caused by hyperphosphorylated tau. To further investigate the protective mechanism underlying such proteolytic modification, we turned to a driver line *202508-Gal4*, which controls the transgene expression in a small subset of GABA neurons for detailed phenotypic analysis (Fig. 4a). Using a dendritic marker (UAS-Denmark) in combination with an axonal marker (UAS-synaptotagmin-GFP), we identified that somas of *202508-Gal4*-driven cells surround the antennal lobe, and axons innervate the antennal lobe (Fig. 4b).

We examined the cell morphology of *202508* > *E14FL* and *202508* > *E14421*, which were marked by co-expressed CD8-GFP. The pattern of GFP labeling confirmed that neurons expressing these two isoforms are grossly comparable to lacZ control; however, immunolabeling of pan-tau revealed a discrepancy in tau protein distribution between E14FL and E14421 in which E14FL proteins were present throughout the soma and neuronal process, whereas E14421 proteins seemed largely devoid from the synapse (Fig. 4c). A similar difference was also observed between WTFL and WT421 (Fig. 4c). In addition, using a commonly employed phospho-tau antibody (AT8)^56^, such difference was also observed between WTFL and WT421, whereas the pseudophosphorylated tau isoforms were all negative for the staining^57^ (Fig. 4c, d). To validate the difference of tau protein distribution among full-length and truncated forms, we co-expressed synaptic marker syt-GFP in E14FL/WTFL- or E14421/WT421-expressing neurons. Immunolabeling of pan-tau confirmed synaptic localization of E14FL with partial colocalization with syt-GFP, which phenomenon was not significant in E14421 (Fig. 4e, f).

To confirm the immunostaining data, we isolated the neuronal fractions from 10-day-old flies and analyzed the enrichment of tau in nucleus/high-density and synaptosome fractions. Consistent with the immunolabeling results, we found that E14FL is relatively more enriched in synaptosomes compared to the nucleus fraction (Fig. 4g, h), whereas E14421 is less abundant in synaptosomes (Fig. 4g, h). The different neuronal distribution of the E14FL and E14421 thus coincide with the severity of tau-induced toxicity described above. Together, our data suggest that the impaired synaptic function observed in hyperphosphorylated tau may be ameliorated due to D421 cleavage at E14 could reduce its accumulation at the synapses.

### D421 truncation of hyperphosphorylated tau protects neurons against the formation of axonal spheroids and aberrant actin accumulations

**Figure 5:**
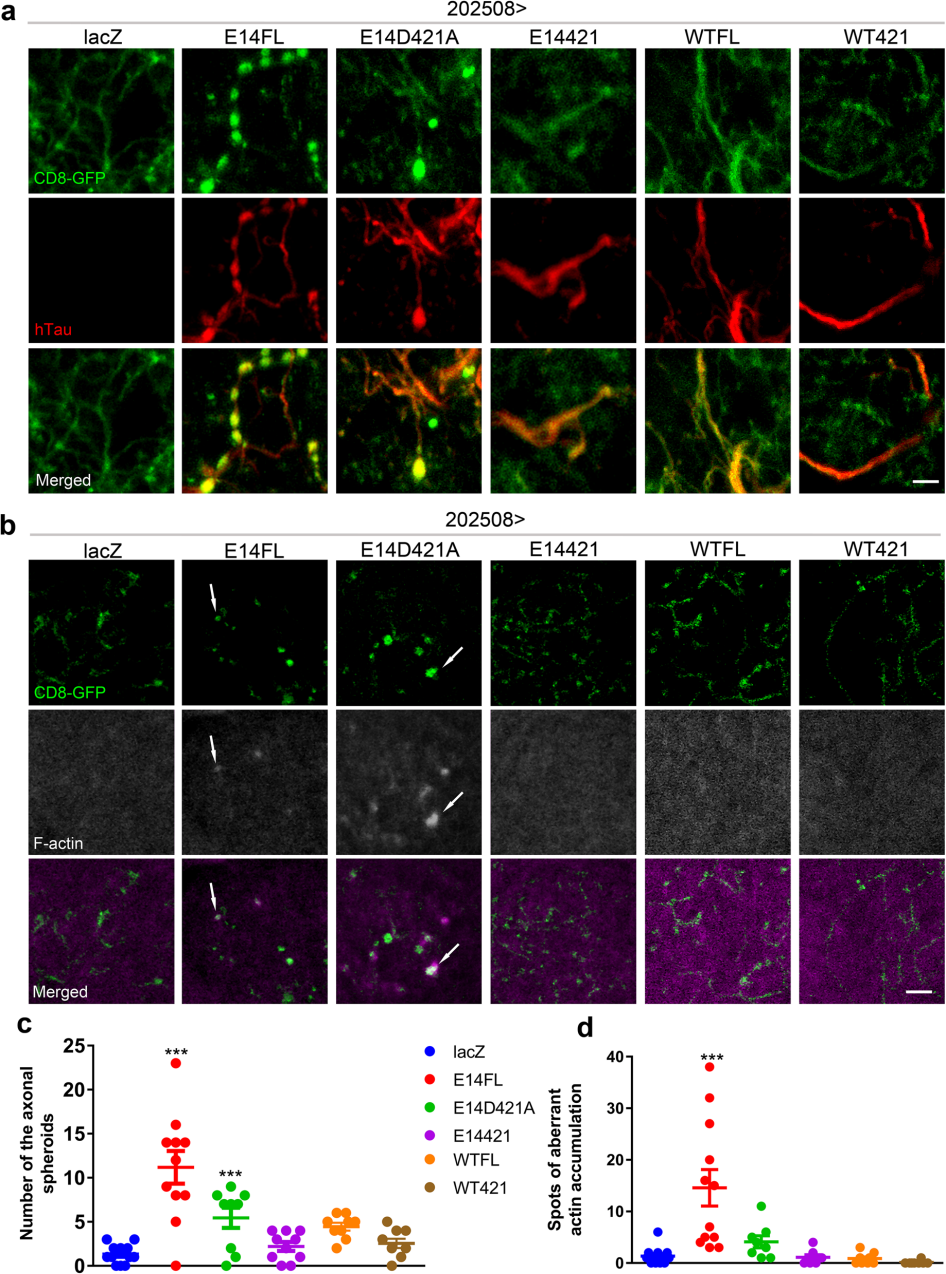
Full-length, but not truncated, hyperphosphorylated tau induces axonal spheroids and aberrant actin accumulations. **(a, b)** Confocal representative images of 10-day-old adult brains expressing CD8-GFP (green) and lacZ (control), or the indicated tau isoforms under the control of *202508-Gal4*. Samples are immunolabeled with anti-hTau (**a**, red) or stained with phalloidin to label F-actin (**b**, magenta in merged panels are converted to gray pseudocolor in F-actin panels for clarity). White arrows in **(b)** mark the aberrant actin accumulations. Scale bars: 5 μm. **(c, d)** Quantification of the numbers of axonal spheroids and aberrant actin accumulations from samples in **a** and **b**, respectively, ***p < 0.001 (8 to 12 brains are analyzed in each genotype, one-way ANOVA with Bonferroni multiple comparison tests).

To better dissect the pathogenic effect of E14FL in axons and terminals, we examined 202508 neurons closely and identified the presence of spheroids in axons, which phenotype was not found in neurons expressing E14421 (Fig. 5a). Previous studies have shown that these hallmark axonal deformations are coupled with the aberrant accumulation of cytoskeletal proteins^32,58^. Therefore, we examined actin in E14FL, and indeed, with F-actin labeling^33^, we found that the expression of E14FL generated some aberrant actin accumulations when the axonal spheroid formation was noted (Fig. 5b and Supplementary Fig. S4a). Some of the aberrant actin accumulations colocalized with axonal spheroids, indicating intracellular accumulation of F-actin in tau-expressing neurons (Fig. 5b), which is consistent with a prior clinical study^34^. Quantification of axonal spheroids and the aberrant actin accumulations revealed that expression of E14FL or E14D421A could induce the formation of axonal spheroids (Fig. 5c), but only E14FL induced aberrant actin accumulations that were significantly more abundant than in the E14421 group (Fig. 5d). Moreover, we detected the number and size of these structures were increased in aged E14FL flies (see Supplementary Fig. S4b online), consistent with a clinical study^59^.

### Phosphorylated tau aggregates are not responsible for the observed toxicity

**Figure 6:**
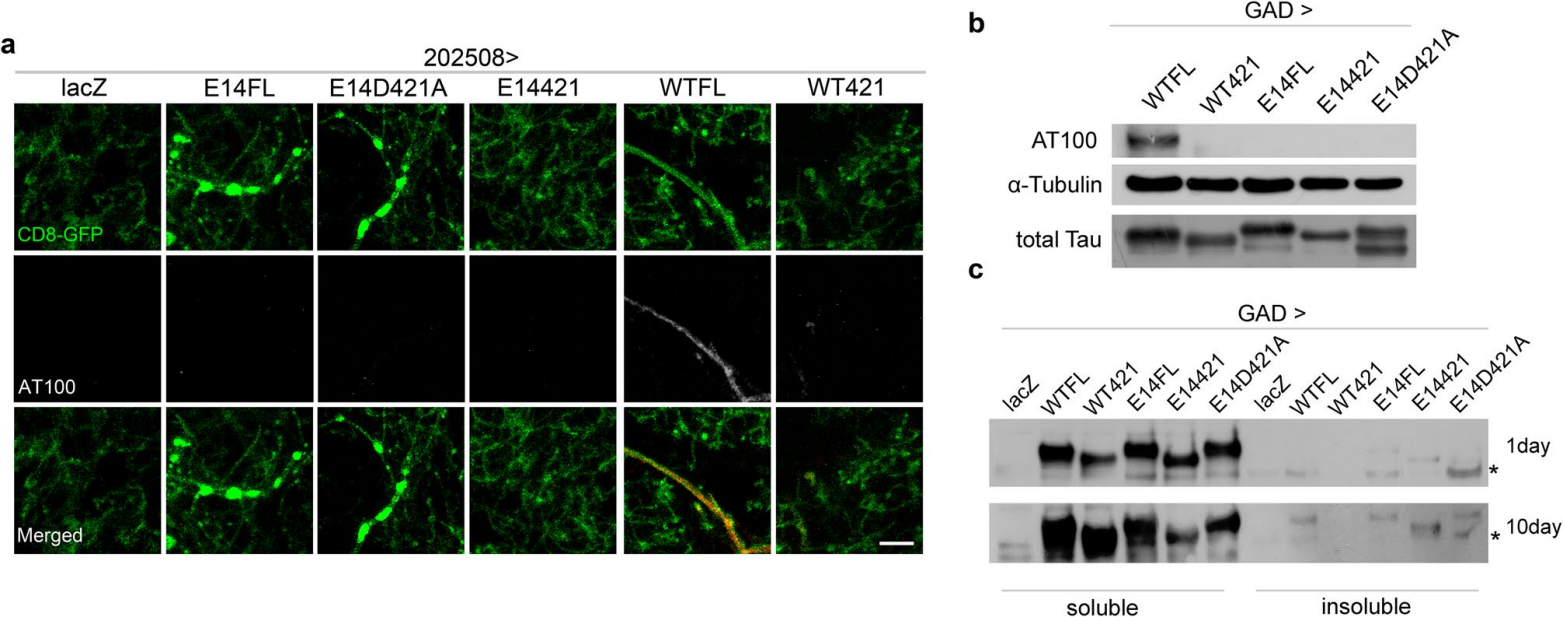
The axonopathy induced by hyperphosphorylated full-length tau dissociates from paired helical filaments and sarkosyl-insoluble aggregation. **(a)** Confocal representative images of 10-day-old adult brains expressing CD8-GFP (green) and lacZ (control), or the indicated tau isoforms under the control of *202508-Gal4*. Samples are immunolabeled with anti-phospho-Tau antibody (AT100, magenta, used for detecting PHF). The middle panels of AT100 staining use gray pseudocolor for clarity. Only the WTFL axons, which do not form axonal spheroids, is positive of AT100 signals. Scale bar: 10 μm. **(b, c)** Western analyses of **(b)** phospho-tau protein (AT100) levels from 10-day-old fly heads of the indicated tau isoforms under the control of *GAD-Gal4*. The blot is stripped twice to sequentially re-probe α-Tubulin and total tau to serve as loading controls. The images are cropped from the same blot, and the full-length blot refers to Figure S13. **(c)** Sarkosyl extraction of tau protein in soluble and insoluble fractions from either 1-day-old (upper panel) or 10-day-old (lower panel) fly heads expressing the indicated tau isoforms under the control of *GAD-Gal4*. The asterisks in **(c)** indicate non-specific bands. The images are cropped from the same blot, and the full-length blot refers to Figure S14.

Because the major pathological hallmark of tauopathy is tau aggregation, thus we asked if the observed axonopathy and the toxicity is related to tau aggregation formation. First, we used AT100, a commonly used readout of tau aggregation that detects the phosphorylated residues within the proline-rich domain (T212/S214/T217) of tau to investigate the link between aggregation and pathogenesis involving hyperphosphorylated tau^44^. we observed little, if any, signal in E14FL, E14D421A, E14421, or control neurons (Fig. 6a). However, we found some positive signals in the axons of WTFL (Fig. 6a), suggesting phosphorylated tau aggregations were formed within the axons of WTFL without inducing significant axonopathy. To further explore whether the aggregation formation might associate with the observed pathophysiological defects, we used sarkosyl extraction to isolate insoluble tau aggregations^44^. Despite the disparate toxic effect between E14FL and E14421, we found that both tau variants developed minimum sarkosyl-insoluble tau aggregates at 1-day adult brains. When examining the brain extracts at 10-day old, all the expressed tau variants, with the exception of WT421, presented detectable insoluble tau aggregates (Fig. 6b, c). Together, these results suggest that the phosphorylated tau aggregations are not the major pathogenic entity responsible for the observed pathophysiological differences between E14FL and E14421.

## Discussion

The dominant view concerning tau phosphorylation and its impact is that hyperphosphorylation under pathological conditions prompts the modified proteins to dissociate from microtubules, causing aberrant tau aggregation and neurodegeneration. Contrary to this view, here we present results showing some commonly phosphorylated Ser/Thr residues found in diseased brains that being modified to phospho-mimicking or non-phospho-modifiable variants are equally toxic. Furthermore, the truncation of full-length tau at D421 can ameliorate the neurotoxic effects caused by tau hyperphosphorylations. Together, these data demonstrate an intricated interplay of tau PTMs apart from kinase modifications contributes to tauopathy.

A previous report showed that pseudophosphorylation of tau^T205^ ameliorates the toxicity of tau^14^. In our study, pseudophosphorylation of the same residue was included in the E14 construct, but—in combination with the 13 other phospho-modifications—it did not prevent tau toxicity. This data suggests that a single factor does not entirely determine the cytotoxic impact, but instead a collective effect of tau PTMs that likely act in a temporally and spatially dynamic manner. We were surprised that the expression of tau transgenes with the same 14 Ser/Thr residues mutated to Ala, which prevents phospho-modification and thus imitated hypophosphorylation, also resulted in cytotoxicity that was similar to, if not stronger than, the phospho-mimicking counterpart in all tested assays. This finding contradicts a previous fly model of tauopathy in which the expression of identical AP modifications in 0N4R isoform only reported mild phenotype^38^. The discrepancy may reside in different tau isoforms being used, or the protein expression levels as increasing the expression of 0N4R tau with AP mutations could exacerbate cytotoxicity^38^. Indeed, it was found that the expression of 0N4R tau AP mutant could disrupt the axonal trafficking of transport vesicles, and the resulted damage was more severe than WT and E14 ^60^. Importantly, the toxicity observed in our model supports the argument that blocking the phosphorylation of specific sites within these 14 targeted Ser/Thr residues in APFL may also be harmful to normal tau^60,61^.

We found the truncation at D421 (E14421) could revert E14FL-induce pathophysiological deficits implying the option of cleaving this site may have an exceeding impact on tau toxicity than the phospho-modification of single sites. Studies on tau truncation at D421 and its impact on the protein's toxicity have been conducted through clinical observations^8,62,63^ and transgenic approaches^21,44^. It was found that D421-truncated tau is enriched in the neurofibrillary tangles in AD brains^8,62^. Furthermore, in a mouse model expressing full-length human tau with a mutation related to familial dementia, endogenous truncations were identified^21,44^, suggesting proteolytic cleavage of tau is intrinsically activated. Although the presence of truncated tau is associated with the progression of AD^8^, whether this innate proteolytic process intends to protect or destruct the neuron remains unclear. We found that the truncation of tau at D421 ameliorated neurotoxicity caused by the protein expression of a set of Ser/Thr residues that were either pseudophosphorylated or unable to be phosphorylated. A fly model expressing 0N4R isoform that is truncated at the corresponded full-length tau (2N4R) D421 site reported enhanced cytotoxicity compared to wild-type 0N4R tau^24^. As the expression of either WTFL or WT421 in our model did not show abnormality in our assays, the discrepancy could be due to the absence of the N-terminus in tau 0N4R isoform, which may have a role in modulating tau toxicity. An early study showed that the very N-terminal end of tau might be neurotoxic, whereas an extended portion of N-terminus, covering the 2N region, maybe neuroprotective^64^. Others found that this portion of tau could regulate α-tubulin acetylation and microtubule stabilization^65,66^. In any case, the effect of D421-truncation on other endogenous tau isoforms or PTM variants warrants further investigations. It is also noteworthy that while the truncation of full-length tau at D421 translated into a distinct distribution of tau variant in the axonal terminal, the functional benefit towards the phospho-mimicking E14. In supporting this, a study found that the knock-in mutant with an abolished D421 cleavage site of mouse tau showed memory/synaptic plasticity defects^67^. Together with our result, it raises a possibility that, during the pathogenesis of tauopathy, if the hyperphosphorylated tau can be truncated at D421 at an earlier time point, it may reduce tau-induced neurotoxicity. Considering that neurons can withstand the pathological burdens for a long time before death, it is reasonable to consider that some endogenous modifications, including truncation at D421, protect neurons from the toxic insult by hyperphosphorylated tau.

The seemingly protective effects of tau truncation at D421 raises the question of what kind of underlying mechanism for this proteolysis to neutralize hyperphosphorylation-induce toxicity might be. Indeed, the effect of interactions between different PTMs is a challenging question for which little evidence is available^18,63^. In terms of the relationship between phosphorylation and truncation, while the proteolytic cleavage at D421 has been considered an early event preceding phosphorylation^25^, a study on the temporal sequence of tau PTMs revealed that tau phosphorylation occurs before proteolytic cleavage at D421^21^. Therefore, whether there is a causal link between these two modifications is still under debate^21,25,44,62^. From the experiments with E14FL and the corresponding D421-truncated mutant E14421, we found that while their expression levels are comparable, E14421 showed much less toxicity than E14FL. The most pronounced difference was associated with changes in tau’s distribution within neurons, which might depend on specific molecular interactions of tau to settle its location^68^. Because the behavioral results showed that E14FL flies display activity deficits similar to those of flies expressing *shibire*, which hinders synaptic transmission, it is tempting to speculate that the synaptic enrichment of hyperphosphorylated tau might be detrimental to synaptic transmission^69^. Therefore, it is reasonable to postulate the limited distribution of E14421 in comparison with E14FL reduced its chance to interact with synaptic molecules, and less prone to induce axonopathy. Indeed, E14FL, but not E14421, induced the formation of axonal spheroids and aberrant actin accumulations, which are neuropathological indications of impaired neurotransmission^70,71^.

A clinical study showed that the axonal spheroids were occasionally associated with the phosphorylated tau aggregates^58^. In this study, while we found the phosphorylation of WTFL at AT8 and AT100 epitopes and the development of tau aggregation, it failed to induce axonal spheroids, suggesting that phosphorylated tau aggregations are not responsible for such structural deform. An earlier study reported that expressing disease-linked human tau mutant could induce toxicity in *Drosophila* without forming neurofibrillary tangles^37^. In agreeing with this finding, although we detected only a small amount of sarkosyl-insoluble tau, an indication of filamentous tau, in both E14FL and E14421, however, the later did not show an evident pathophysiological defect. Previously it was reported that the presence of D421-truncated tau is associated with the aggravated tau aggregation in a *Drosophila* model^24^. Interestingly, we did not observe that the truncation can increase aggregation propensity, which is similar to another study that showed the truncated tau protein was mainly soluble^21^. Moreover, its proportion to total tau under different tauopathy backgrounds showed no difference^21^, suggesting that insoluble tau might not be the most pivotal factor in tauopathy.

Because the only difference between E14421 and E14FL is the deletion of the last 20 amino acids to the C-terminus of tau, raising a possibility that additional modification(s) within this domain, or through the interaction with other modifiers, might be pivotal in tau-mediated neurotoxicity. Additional studies are required to disclose the relationship between different PTMs variants of tau and their protein interaction properties in the future.

## Supplementary information

Supplementary Information.

## Data Availability

All data generated or analysed during this study are included in this published article.
