# Supplementary material for: Cleavage of human tau at Asp421 inhibits hyperphosphorylated tau induced pathology in a Drosophila model
Source: Sci Rep. 2020 Aug 10;10:13482. doi: 10.1038/s41598-020-70423-1 (PMC7417559; doi:10.1038/s41598-020-70423-1)
Supplement: Supplementary file 1 — Supplementary Information. [file 41598_2020_70423_MOESM1_ESM.pdf]

Supplementary information

Cleavage of human tau at Asp421 inhibits hyperphosphorylated tau induced pathology in a *Drosophila* model

[1]Hao Chi, [1]Lee Sun, [1]Ren-Huei Shiu, [2]Rui Han, [1]Chien-Ping Hsieh, [3]Tzu-Min Wei, [2,3,4]Chung-Chuan Lo, [3,5]Hui-Yun Chang, [*1,4,6]Tzu-Kang Sang

Affiliations:

[1]Institute of Biotechnology, National Tsing Hua University, Hsinchu 30013, Taiwan.

[2]Institute of Bioinformatics and Structural biology, National Tsing Hua University, Hsinchu 30013, Taiwan.

[3]Institute of Systems Neuroscience, National Tsing Hua University, Hsinchu 30013, Taiwan.

[4]Brain Research Center, National Tsing Hua University, Hsinchu 30013, Taiwan.

[5]Department of Medical Science, National Tsing Hua University, Hsinchu 30013, Taiwan.

[6]Department of Life Science, National Tsing Hua University, Hsinchu 30013, Taiwan.

Contact information:
Hao Chi, email: truthbj8899@gmail.com; Lee Sun, email: calvin.leesun@gmail.com; Ren-Huei Shiu, email: sharon01068@yahoo.com.tw; Rui Han, email: hanrui901120123456@gmail.com; Tzu-Min Wei, email: F41027@lolab-nthu.org; Chien-Ping Hsieh, royhsieh129@gmail.com; Chung-Chuan Lo, cclo@mx.nthu.edu.tw; Hui-Yun Chang, huiyun@life.nthu.edu.tw; [*]Tzu-Kang Sang (Corresponding author), Tel: 886-3-5742474. Fax: 886-3-571-5934. Email: tksang@life.nthu.edu.tw

Supplementary figure 1

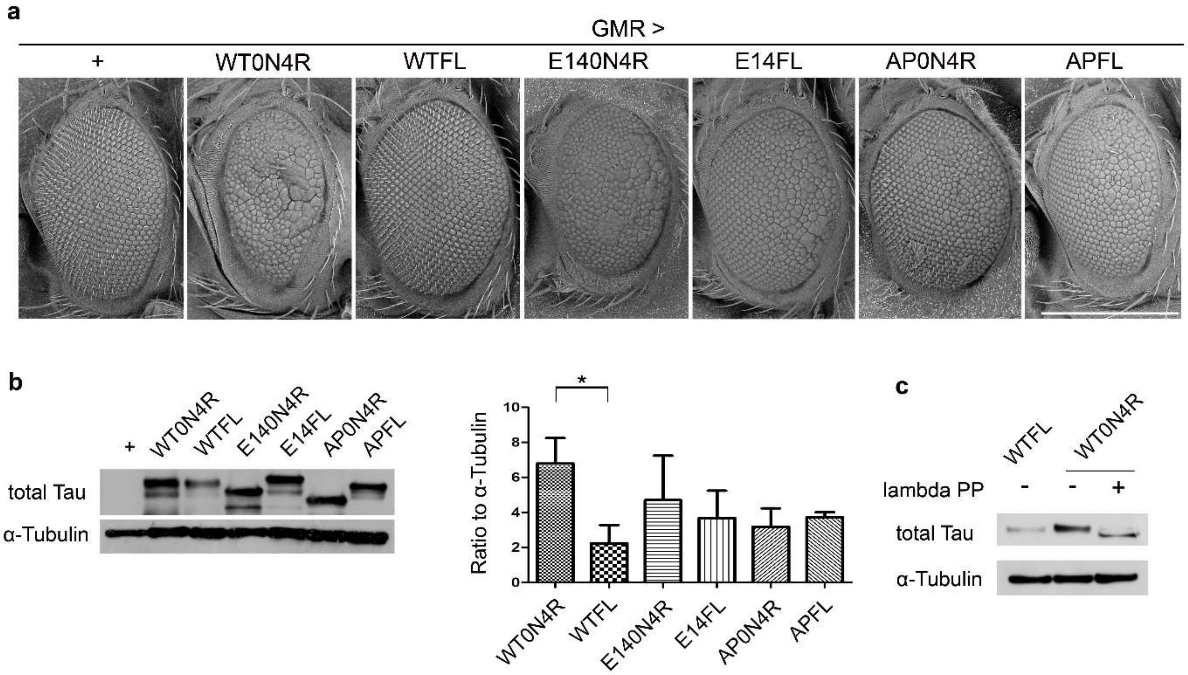

Figure S1. The increased expression levels of wild type tau as compared to the 2N4R counterpart is associated with the severity of the rough eye phenotype. (**a**) SEM images of the compound eyes expressing the indicated tau isoforms under the control of *GMR-Gal4.* All images are taken from 1-day-old adult flies. Scale bar: 300 μm. (**b**) Western analysis of tau protein levels in tissues expressing the indicated tau isoforms under the control of *GMR-Gal4*. The blots are stripped and re-probed with α-Tubulin to serve as a loading control. The ratio values are calculated as "total Tau/α-Tubulin" and presented as mean ± SE. *p<0.05 (n=3, one-tail student *t*-test comparing 0N4R and 2N4R counterparts). The images are cropped from the same blot, and the full-length blot refers to figure S7. (**c**) Western analysis of phospho-modifications of 0N4R tau. Protein lysates from 1-day-old, *GMR-Gal4*-drived 2N4R tau (WTFL), and 0N4R tau with (+) or without (-) Lambda phosphatase treatment. The blot is stripped and re-probed with α-Tubulin as a loading control. The images are cropped from the same blot, and the full-length blot refers to figure S8.

Supplementary figure 2

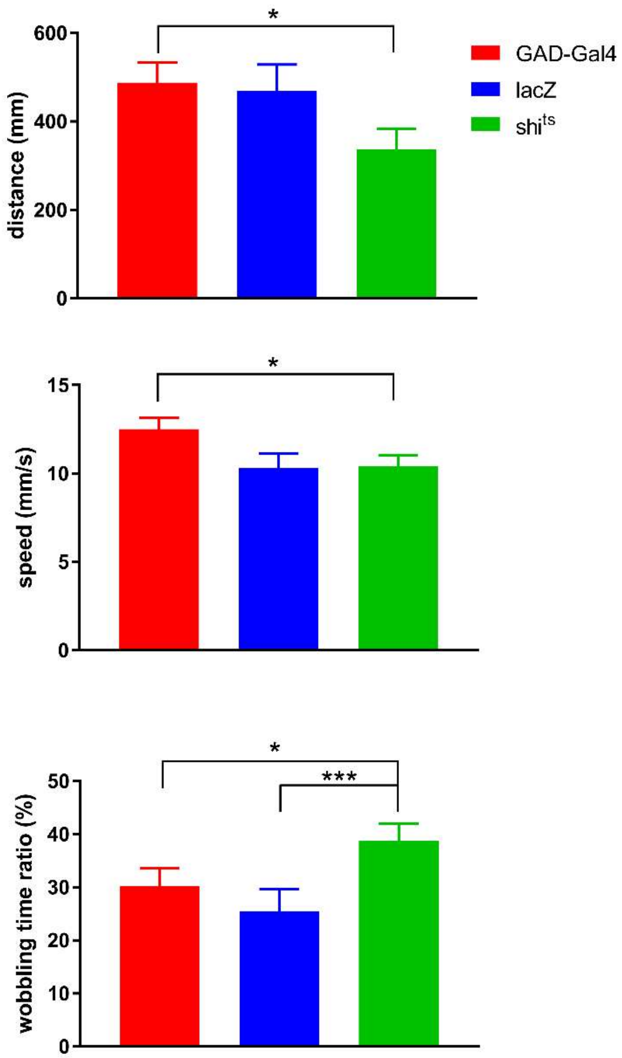

Figure S2. Blocking the neurotransmission by expressing temperature-sensitive *shibire* in GABAergic neurons induces motor impairments. Behavioral analyses of the flies expressing temperature-sensitive *shibire* (*shi^{ts}*) or lacZ control under the control of *GAD-Gal4*. Flies are reared at 18 C° and shift to 30 C° for two hours right before the tests. The index of each analysis includes distance, speed, and wobbling time ratio should refer to Methods for detail. At least 20 flies (1-day-old) of each genotype are tested Values shown represent mean ± SE. *p<0.05, ***p<0.001 (two-way ANOVA with LSD post hoc tests).

Supplementary figure 3

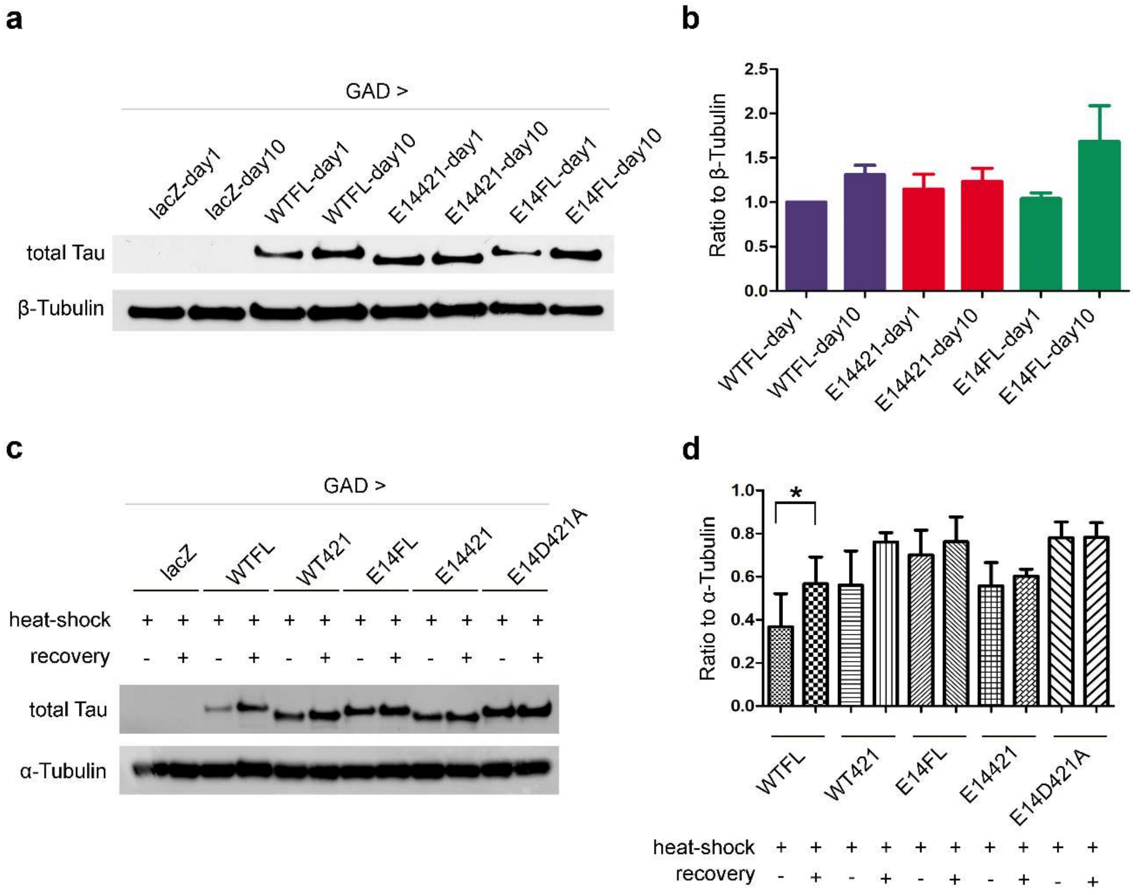

Figure S3. Comparable protein levels among different tau isoforms at different ages and in the protein degradation assay. (**a**) Protein levels of the indicated tau isoforms are measured at two age-matched stages (1-day-old and 10-day-old) by Western analyses. The blots are stripped and re-probed with β-tubulin to serve as a loading control. The images are cropped from the same blot, and the full-length blot refers to figure S9. (**b**) Quantification of replicated results shown in (**a**). The ratio values are calculated as "total Tau/ β-Tubulin" and normalized to the ratio of 1-day-old WTFL (n=4, one-way ANOVA with Bonferroni multiple comparison tests show no significant difference among tau isoforms). (**c**) Protein levels of the indicated tau isoforms are measured by Western analyses after the "heat-shock" to assess tau degradation. The brain lysates are from flies overexpressing *GAD-Gal4* driven tub-Gal80^{ts} and the indicated tau isoform. The flies are placed in 30°C for 2 days after eclosion (+) followed by a 10-days recovery period at 18°C (+) or sacrificed immediately (-). The blot is stripped and re-probed with α-Tubulin as a loading control. The images are cropped from the same blot, and the full-length blot refers to figure S10. (**d**) Quantification of replicated results shown in (**c**). The ratio values are calculated as "total Tau/α-Tubulin",*$P<0.05$. (n=3, one-way ANOVA with Bonferroni multiple comparison tests).

Supplementary figure 4

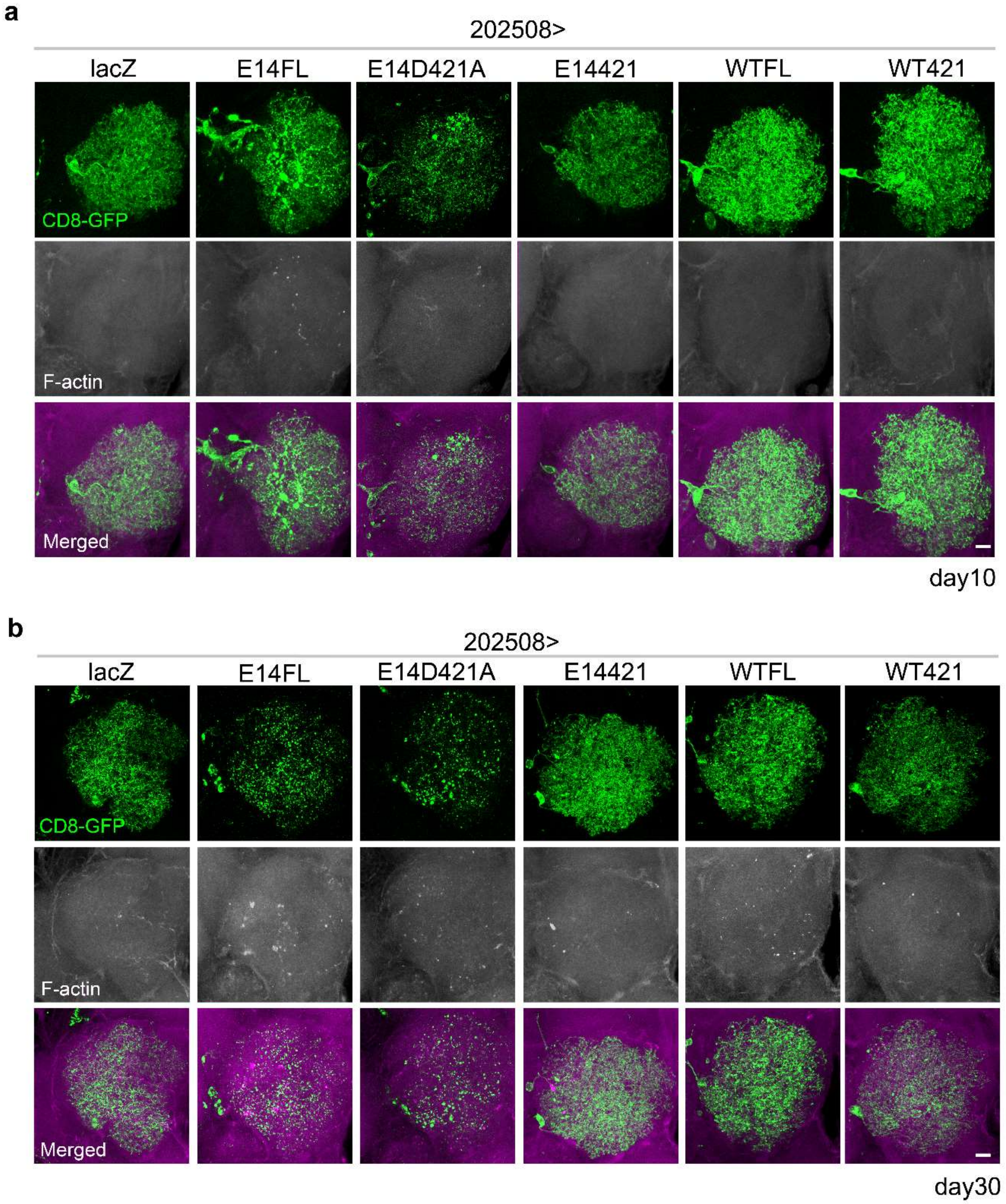

Figure S4. The increase of aberrant actin accumulations in aged flies. Representative confocal images of 10-day-old (**a**) or 30-day-old (**b**) adult brains express CD8-GFP (green), and lacZ (control) or the indicated tau isoforms, driven by *202508-Gal4*. Samples are stained with phalloidin (magenta) to label F-actin (magenta in merged panels are converted to gray pseudocolor in F-actin panels for clarity). Scale bars: 10 μm.

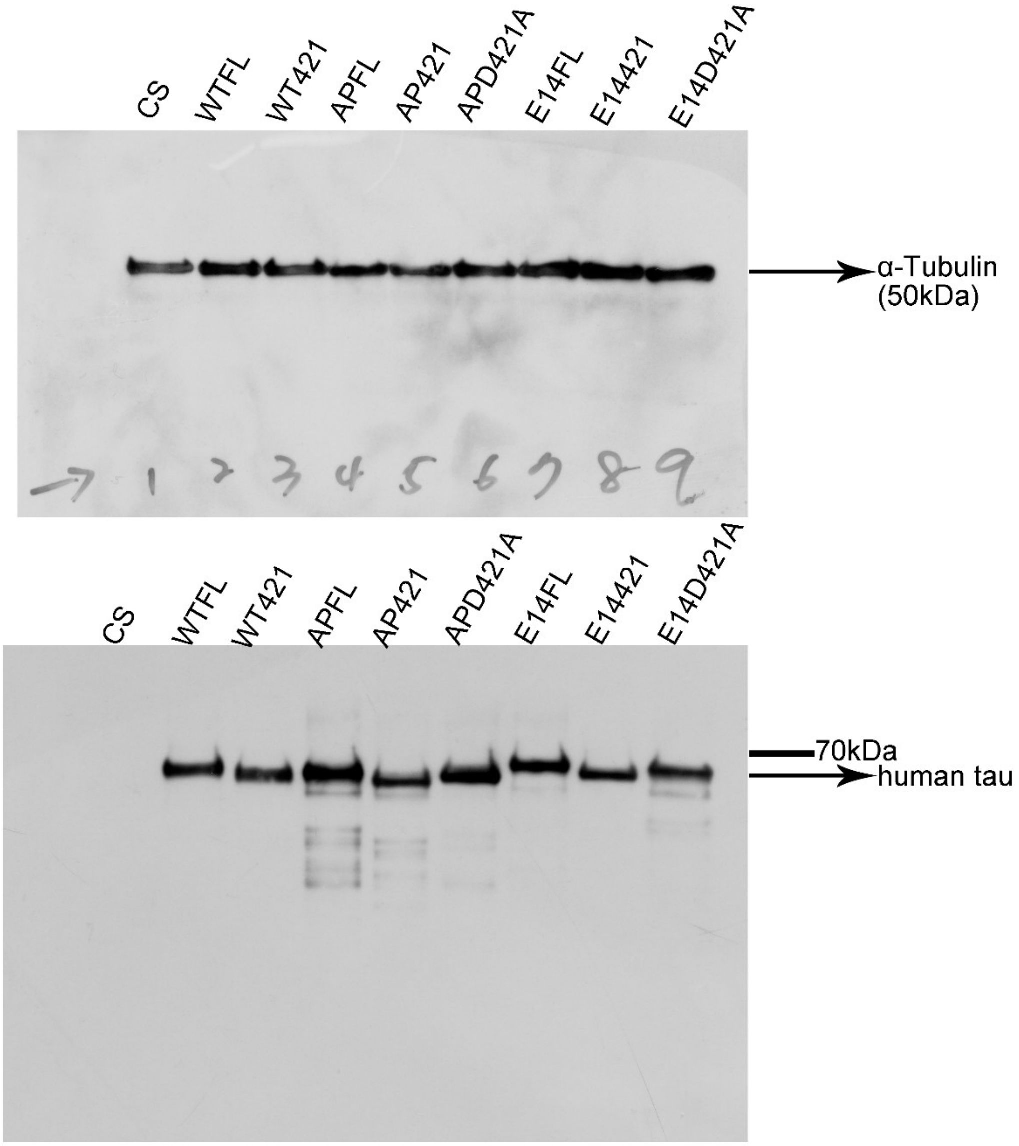

Figure S5. Original Western blot images for Fig. 2d

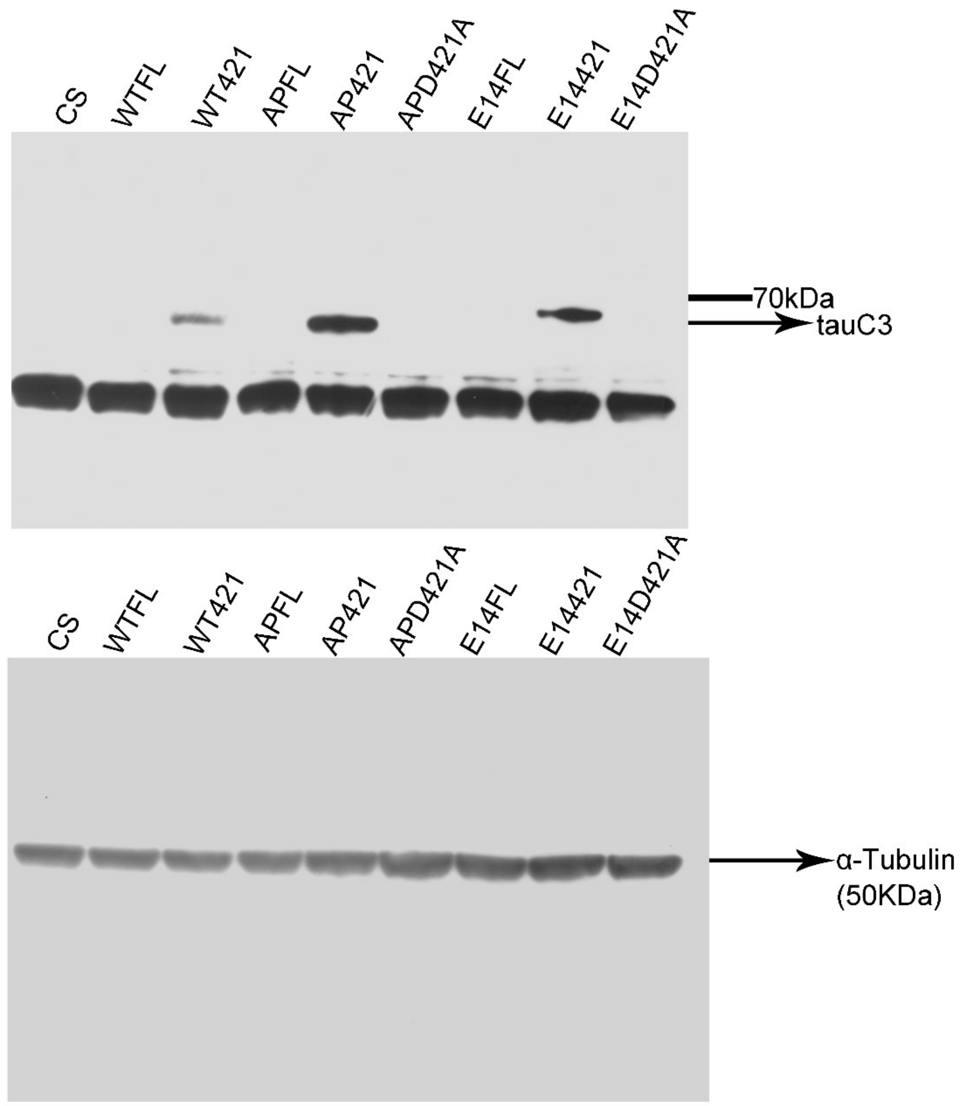

Figure S6. Original Western blot images for Fig. 2e

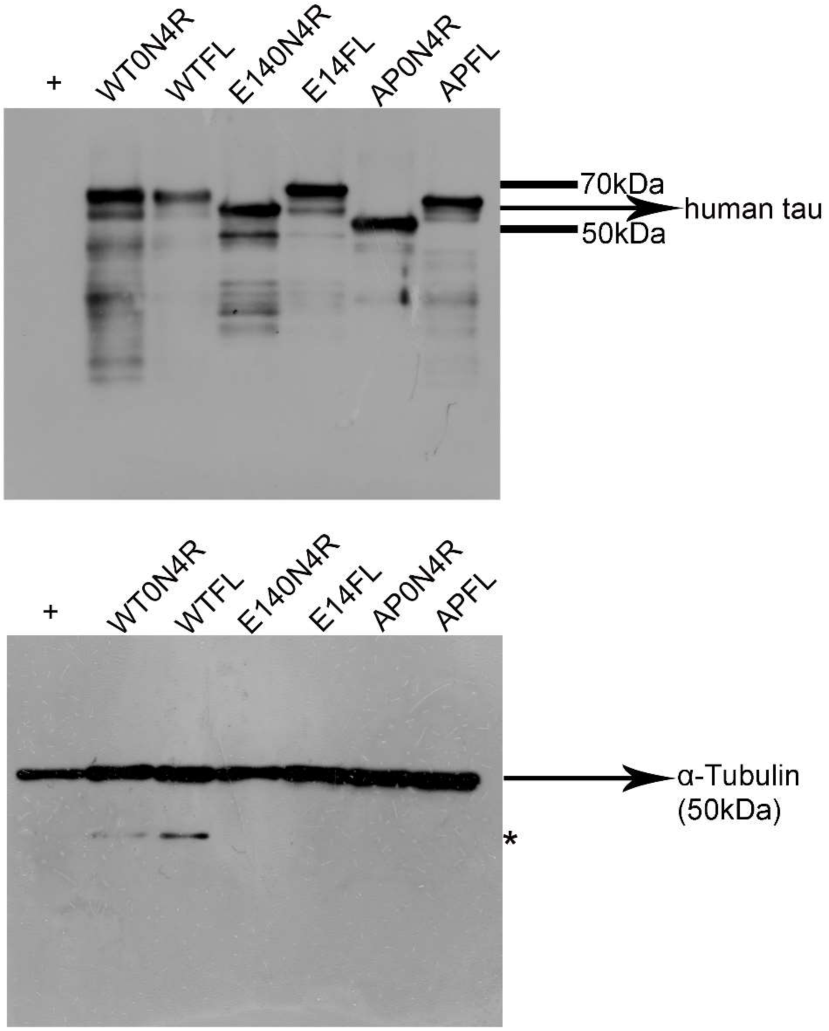

Figure S7. Original Western blot images for Fig. S1b

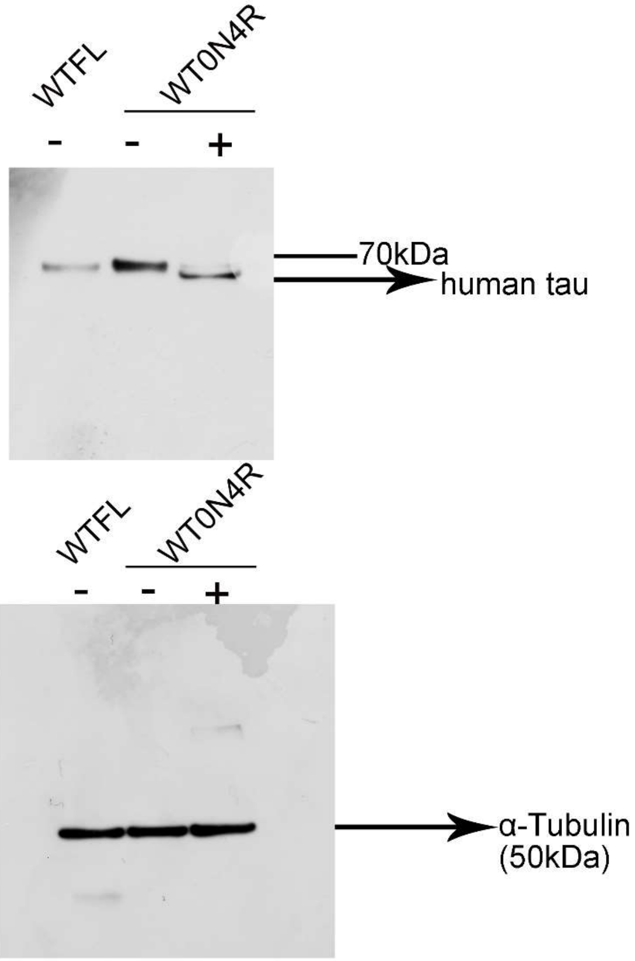

Figure S8. Original Western blot images for Fig. S1c

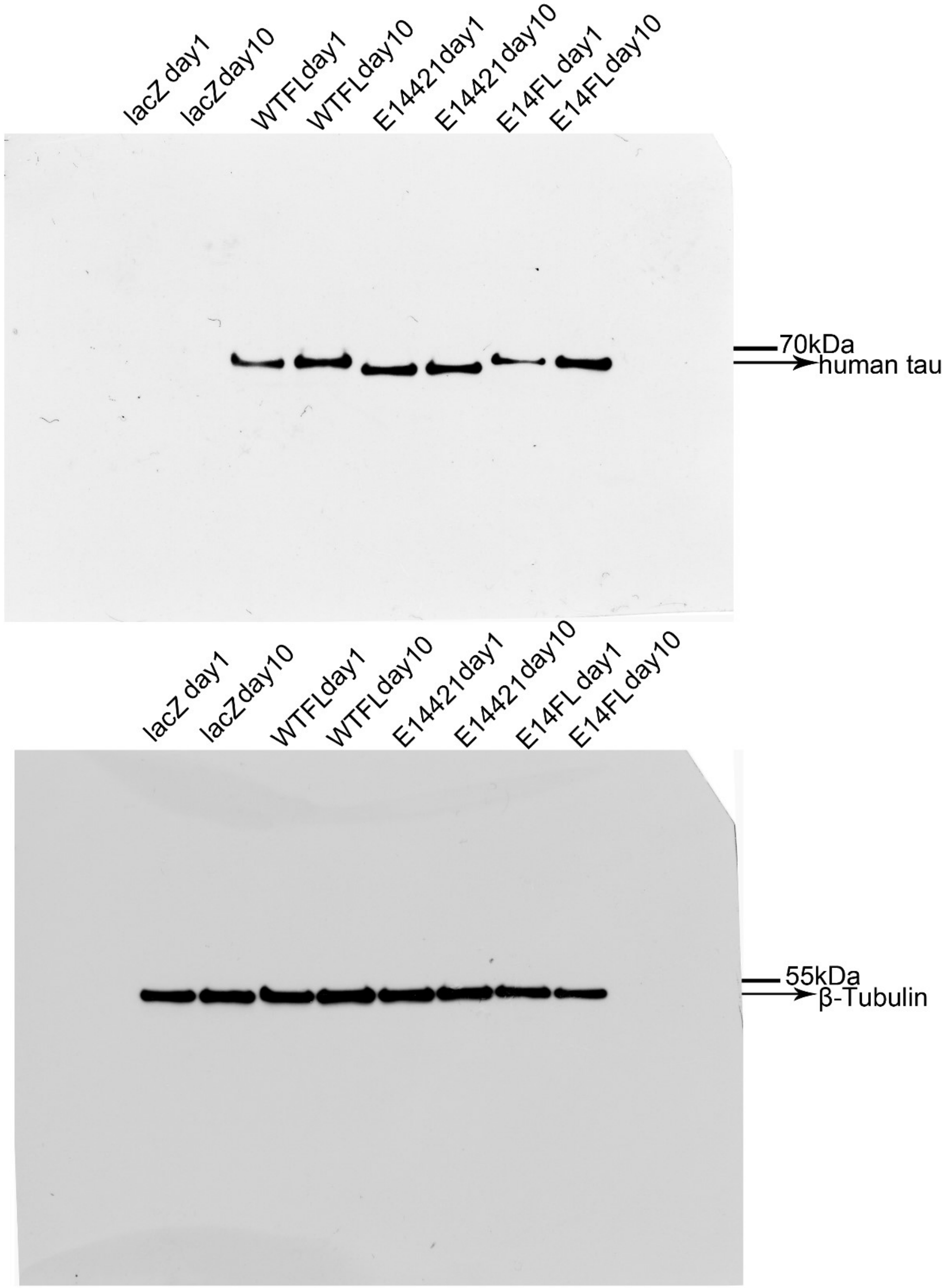

Figure S9. Original Western blot images for Fig. S3a

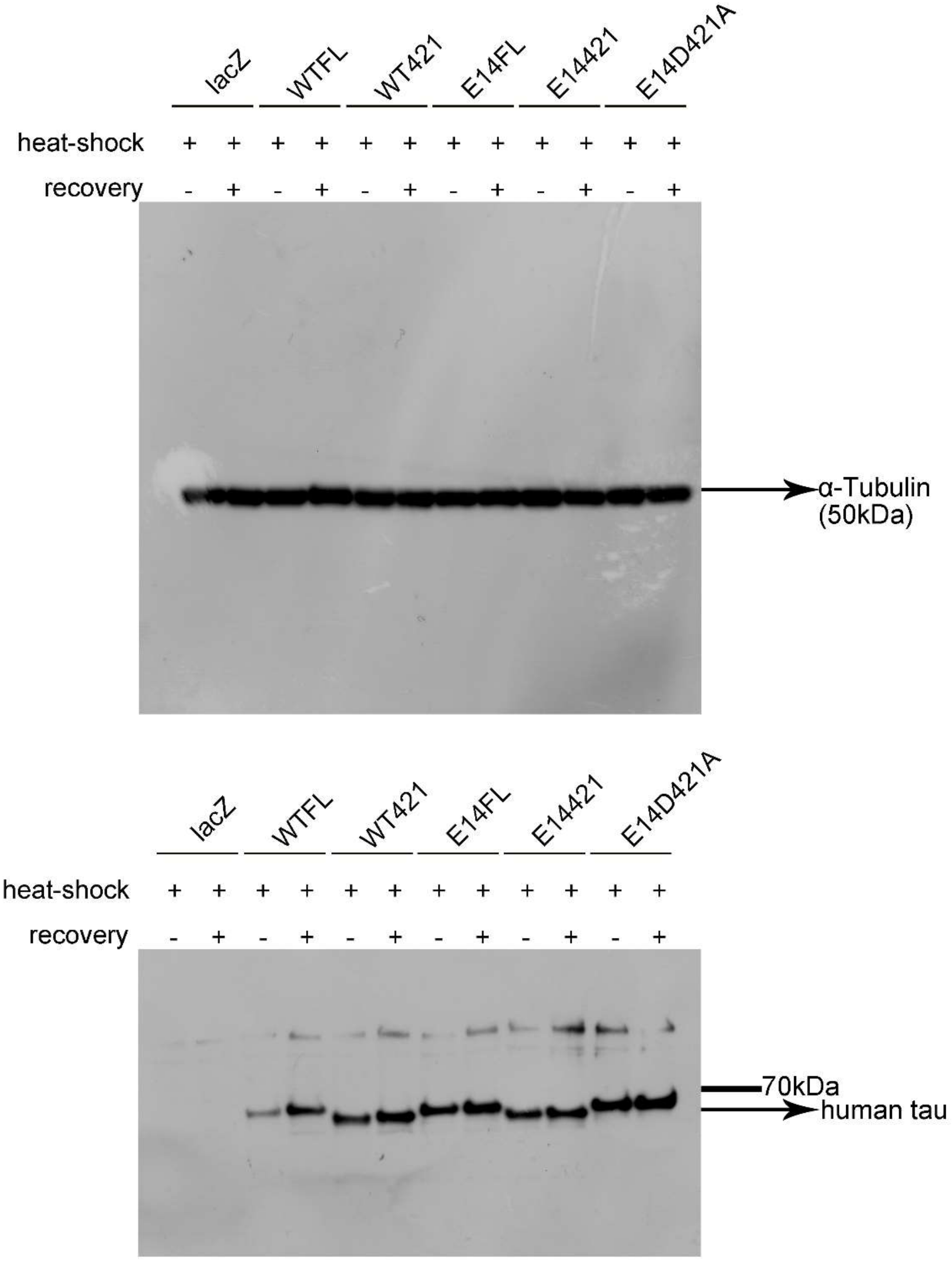

Figure S10. Original Western blot images for Fig. S3c

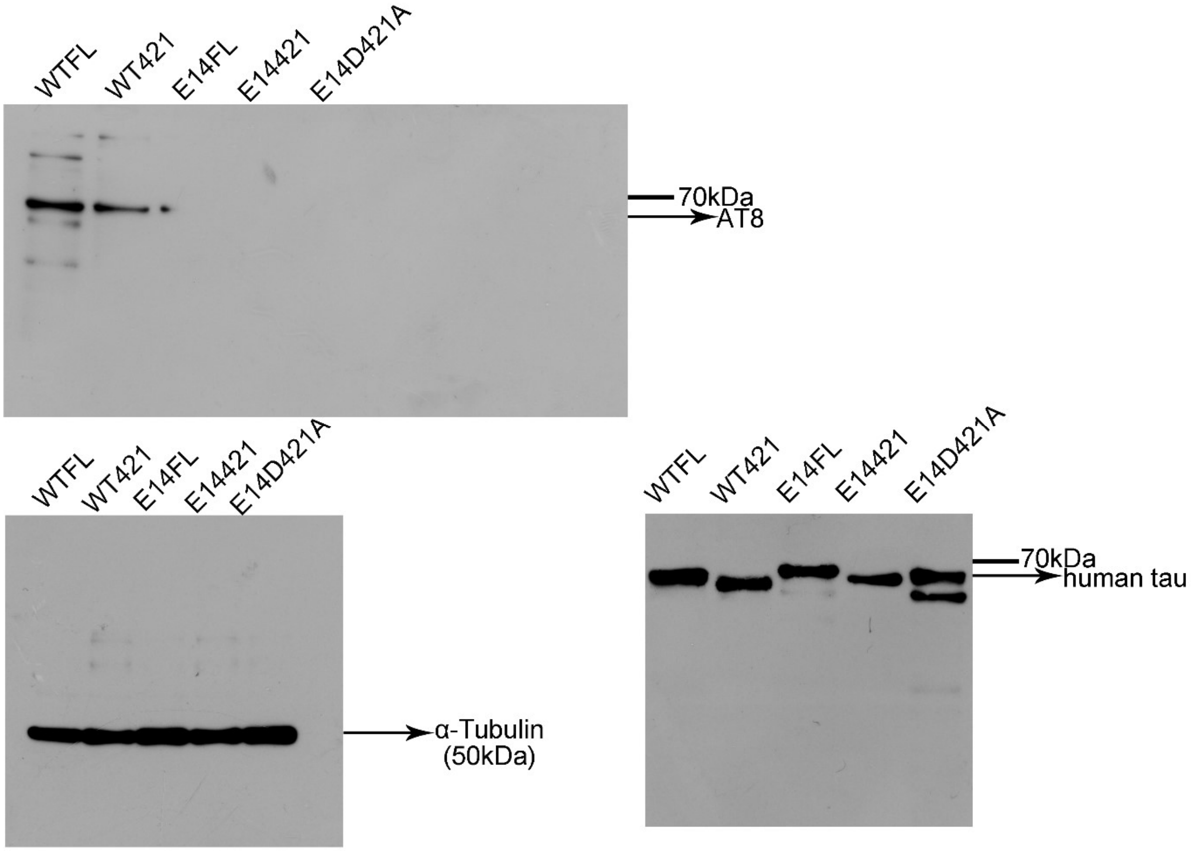

Figure S11. Original Western blot images for Fig. 4d

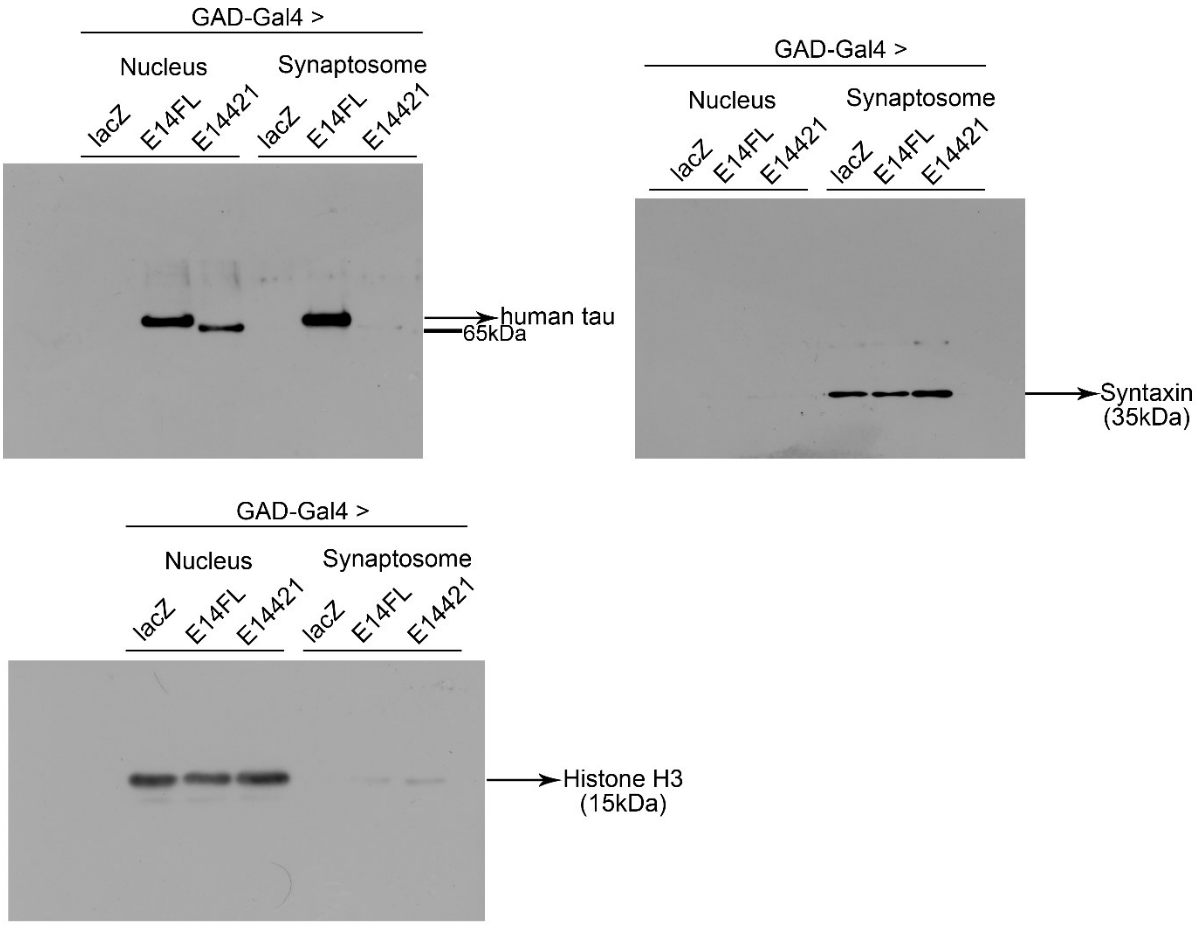

Figure S12. Original Western blot images for Fig. 4g

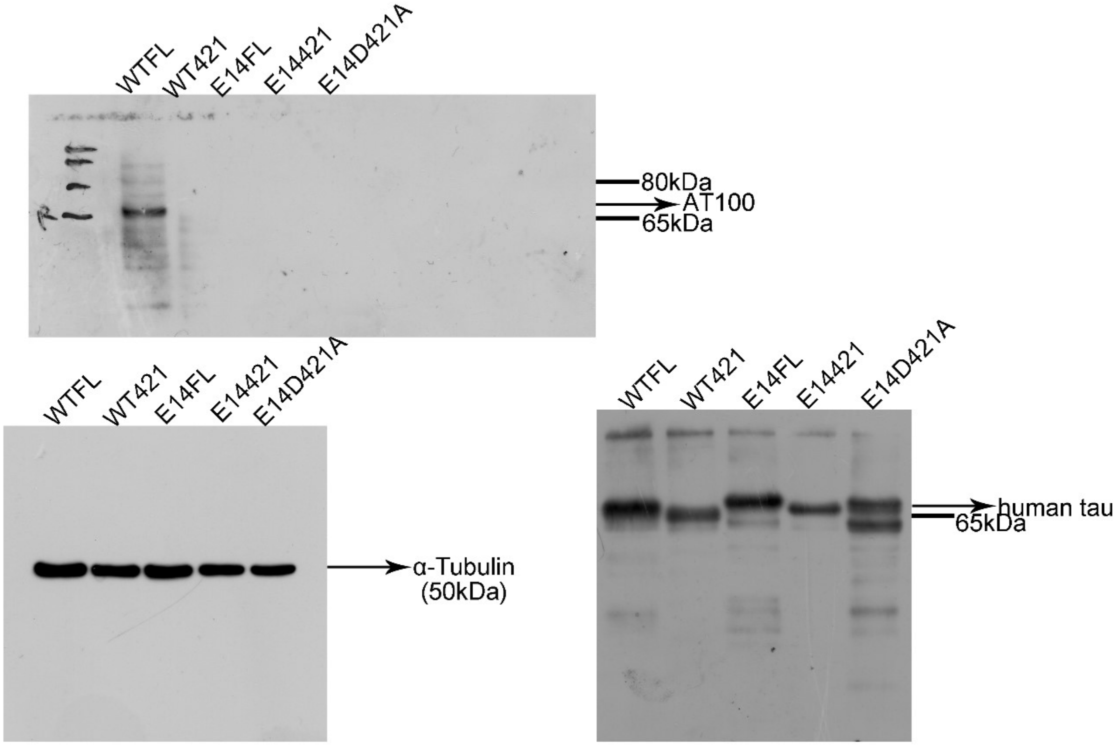

Figure S13. Original Western blot images for Fig. 6b

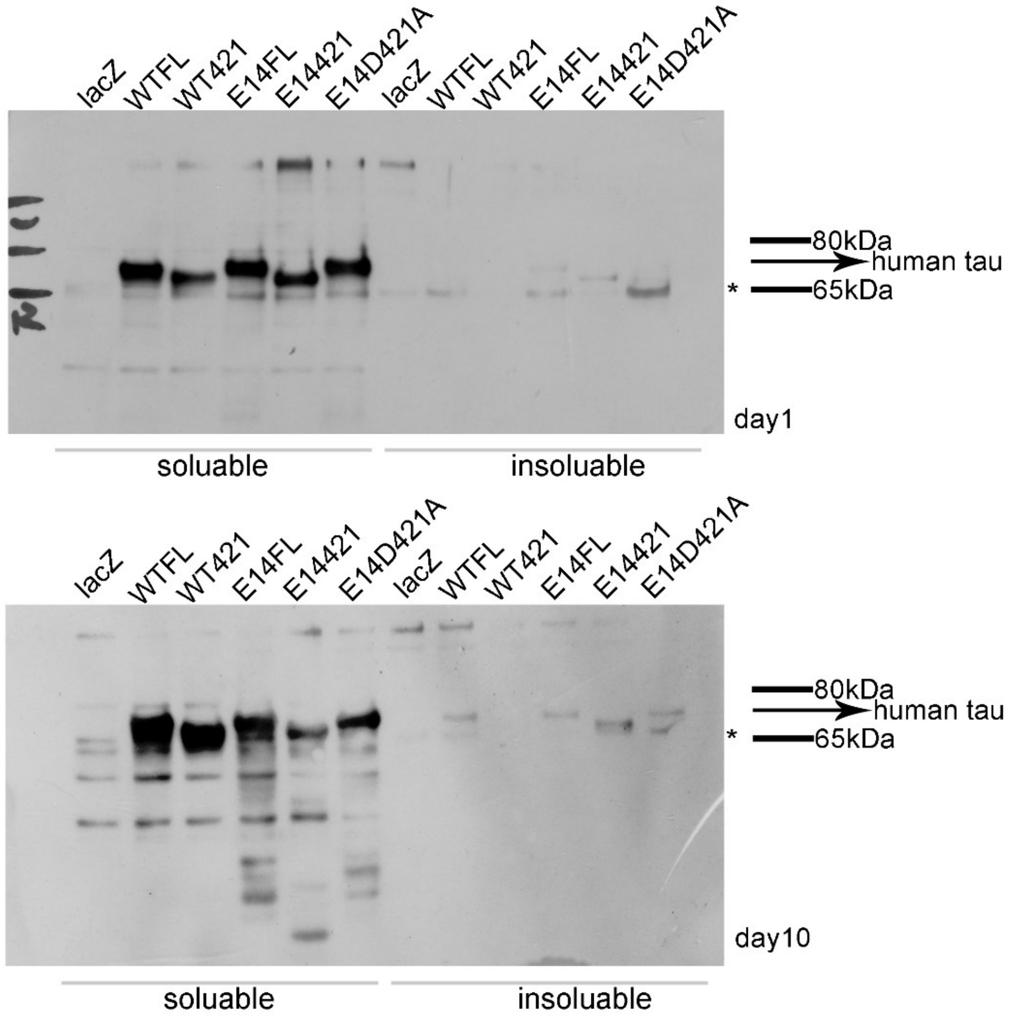

Figure S14. Original Western blot images for Fig. 6c
